# Antigen-dependent modulation of immune responses to antigen-Fc fusion proteins by Fc-effector functions

**DOI:** 10.3389/fimmu.2023.1275193

**Published:** 2023-10-05

**Authors:** Elie Richel, Jannik T. Wagner, Stephan Klessing, Riccardo Di Vincenzo, Vladimir Temchura, Klaus Überla

**Affiliations:** Institute of Clinical and Molecular Virology, University Hospital Erlangen, Friedrich-Alexander-University Erlangen-Nürnberg, Erlangen, Germany

**Keywords:** Fc-fusion proteins, DNA immunization, mice, HIV, SARS-CoV-2, Fc-effector functions

## Abstract

**Background:**

Fc-fusion proteins have been successfully developed for therapeutic purposes, but are also a promising platform for the fast generation and purification of immunogens capable of inducing strong humoral immune responses in preclinical immunization studies. As the Fc-portion of immunoglobulins fused to an antigen confers functional properties of the parental antibody, such as dimerization, binding to Fc-receptors and complement activation, several studies reported that Fc-fusion proteins elicit stronger antigen-specific antibody responses than the unfused antigen. However, dimerization or half-life extension of an antigen have also been described to enhance immunogenicity.

**Methods:**

To explore the role of Fc-effector functions for the immunogenicity of fusions proteins of viral glycoproteins and Fc fragments, the HIV-1 gp120 and the RBD of SARS-CoV-2 were fused to the wild type muIgG2a Fc fragment or mutants with impaired (LALA-PG) or improved (GASDIE) Fc-effector functions.

**Results:**

Immunization of BALB/c mice with DNA vaccines encoding gp120 – Fc LALA-PG induced significantly higher antigen-specific antibody responses than gp120 – Fc WT and GASDIE. In contrast, immunization with DNA vaccines encoding the RBD fused to the same Fc mutants, resulted in comparable anti-RBD antibody levels and similar neutralization activity against several SARS-CoV-2 variants.

**Conclusion:**

Depending on the antigen, Fc-effector functions either do not modulate or suppress the immunogenicity of DNA vaccines encoding Fc-antigen fusion proteins.

## Introduction

Fc-fusion proteins, characterized by the fusion of a protein domain of interest to a functional immunoglobulin G (IgG) Fc fragment, have been developed for the therapy of a number of different diseases including auto-inflammatory conditions or enzyme deficiencies [reviewed in (1, 2)]. Anti-drug antibodies may limit the efficacy of some of these therapeutic Fc-fusion proteins. Antigen-Fc (Ag-Fc) fusion proteins have also been explored for their potential to enhance the immunogenicity of antigens. Due to the presence of a functional Fc-portion, the overall protein retains specific biological and pharmacological properties commonly shared with IgGs. Indeed, the ability of the Fc-portion to interact with the host-cells through the neonatal Fc-receptor (FcRn) has been shown to confer an increased stability and extended half-life to the protein of interest by FcRn recycling mechanisms (3, 4). In addition, Fc-fusion proteins retain the property of IgGs to interact with Fc-gamma receptors (FcγRs) and therefore have the potential to trigger antigen uptake via FcγRs-expressing antigen presenting cells (APCs) leading to a higher antigen uptake and presentation to adaptive immune cells in draining lymph nodes (5, 6).

Since protection against viral infection often correlates with high neutralizing antibodies levels (7, 8), immunization studies with Fc-fusion proteins have been conducted in animal models for investigating their potential in eliciting antiviral antigen-specific antibodies. Immunization of Non-Human Primates using the Human Immunodeficiency Virus type 1 (HIV) gp120 subunit of the Envelope protein (HIV Env) fused to rhesus macaques IgG1 Fc-portion elicited higher anti-gp120 antibody levels in the sera with more potent neutralizing activity than monomeric gp120 immunization (9). Enhanced immunogenecity was also conferred by the Fc fragment in a murine model with Fc-fusion proteins of the Epstein-Barr Virus (EBV) gp350 (10). Due to the recent SARS-CoV-2 pandemic, tremendous efforts have been deployed to generate vaccines and neutralizing monoclonal antibodies against the Spike glycoprotein responsible for the attachment and entry of the virus into host-cells through Spike – human Angiotensin-converting enzyme 2 (hAce2) interaction (11–15). For such purposes, Fc-fusion proteins consisting of the Spike Receptor Binding Domain (RBD) fused to Fc-portion have been rapidly generated and investigated for immunogenicity. Pre-clinical trials in mice and non-human primates revealed that administration of recombinant RBD-Fc fusion proteins led to the elicitation of neutralizing antibodies against SARS-CoV-2 conferring protection against viral challenge (16, 17). In addition, a panel of neutralizing monoclonal antibody was generated early on in the COVID-19 pandemic in humanized mice using an RBD-Fc fusion protein as immunogen (18). Together, these studies indicate that immunizations with bioengineered Fc-fusion proteins consisting of viral antigens coupled to Fc-effector competent Fc fragments induced antigen-specific antibodies in several viral infection models.

However, it is unclear, which of the functional properties of the Fc fragment are responsible for the improved immunogenicity of the Ag-Fc fusion proteins. Dimerization of the antigen, which also occurs through pairing between the Fc fragments of the fusion protein, enhances the half-life of the antigen and additionally increases the avidity for B-cell receptors, thus potentially leading to a better stimulation and proliferation of antigen-specific B-cells compared to the monomer of the antigen. Consistently, recent studies have demonstrated that tandem-repeats of the RBD elicited higher antibody titers with more potent neutralizing antibody activity than monomeric RBD immunizations in the murine model (19–21).

To pinpoint the role of Fc-effector functions for the immunogenicity of Fc-fusion proteins, we generated DNA vaccines encoding viral glycoproteins fused to the wild type murine IgG2a (muIgG2a) Fc fragment or mutants of it with impaired (LALA-PG) or enhanced (GASDIE) binding to Fcγ receptors (FcγRs). Since these point mutants maintain dimerization activity, the role of Fc-effector functions can be separated from potential differences in tertiary or quaternary structures.

## Materials and methods

### Generation of pFuse antigen expression plasmids

An acceptor plasmid consisting of a pFuse expression vector (from InvivoGen, San Diego, CA, USA) encoding for the Fc-portion of a murine IgG2a antibody, therefore omitting the VH and the CH1 domains, was generated by PCR using the primers 5´-ACCAGCTGGACCTGACACTGGACACCGGTTGCAGTTGCTACT- 3’ and 5’-AACCGGTGGCGGAGGATCCGAGCCACGTGGGCCCACAATCAAGCC-3’. The PCR products were designed to bear AgeI restriction sites at each extremity of the amplicons for cloning purposes.

The SARS-CoV-2 RBD of the delta variant Spike (319-541 Aa, Uniprot #P0DTC2) and HIV Consensus clade B gp120 (22) DNA coding sequences were amplified by PCR primers 5’-TTTTCTAGTAGCAACTGCAACCGGTGTCCAGTGTCGGGTGCAGCCCACCGAATC-3’ and 5’-GGCTCGGATCCTCCGCCACCGAAGTTCACGCATTTGTTCT-3’ or primers 5’-TTTTCTAGTGCAACTGCAACCGGTGTCCAGTGTTCCGCCGCCGAGAAGCTGTGG-3’ and 5’-GCTCGGATCCTCCGCCACCGCGCTTCTCGCGCTGCACC-3’, respectively. PCR products were cloned into the acceptor plasmid using the AgeI restriction sites located in the signal peptide sequence of the Fc-portion. An illustration of the respective plasmid maps can be found in **Supplementary Figure S1**. The LALA-PG and GASDIE mutations were then introduced by mutagenesis PCR and cloned into the Ag-Fc expression plasmids by conventional cloning.

Plasmids were transformed and amplified in E.coli XL-Gold™ (Stratagene) bacteria selected in low-salt LB medium (10g/L Tryptone, 5g/L NaCl, 5g/L yeast extract, pH 7.2) supplemented with 25µg/mL of Zeocin® (InvivoGen, San Diego, CA, USA). All genetic sequence modifications were confirmed via Sanger Sequencing (EZ-Seq, Macrogen Europe).

### Mammalian cells culture and transfection

HEK293T/17, TZMbl and hAce2 expressing HEK293T/17 cells were cultured in DMEM medium supplemented with 10% FCS + 2mM Glutamine (all from Gibco, Thermo Fisher Scientific, Waltham, MA, USA) + 1% penicillin/streptomycin (P/S) (= complete DMEM medium) and passaged every 2-3 days according to confluence. Cell lines were grown in a humidified incubator supplemented with 5% CO_2_ at 37°C.

Antigen expression vectors were transfected in HEK293T/17 cells in order to test the expression of the respective immunogens. For that purpose, HEK293T/17 cells were seeded one day prior to transfection in complete DMEM medium. The transfection reactions were set with 2µg of expression plasmid per million cells mixed with polyethylenimine (PEI, Polysciences Inc., Warrington, PA, USA) to a 1:3 mass ratio in DMEM medium + 2mM Glutamine (=D0 medium). Subsequent to a 20min incubation at room temperature (RT), cell media were exchanged for D0 medium and the DNA : PEI mixtures were applied dropwise onto the fresh cell media. Cells were then incubated for 6h in a CO_2_ incubator, after which the media were exchanged for DMEM + 1.5% FCS + 2mM Glutamine (=D1.5 medium). After a 48h incubation in a humidified incubator, the cell supernatants containing the proteins of interest were spun down at 2000xg for 5min to remove cell debris and stored adequately at 4°C.

### Expression analysis by western blot

In order to verify the expression of each Fc-fusion protein, 20µL of cell supernatants were mixed with 20 µL of 2x SDS loading dye in presence or absence of β-mercaptoethanol (β-Me) and boiled for 2min at 95°C. Samples were loaded onto 8% or 10% SDS-PAGE gels and run at 120V for 1h15. The separated proteins were then transferred onto nitrocellulose membranes (GE Healthcare, Healthcare, Little Chalfont, UK), followed by an incubation of 1h under rotation with 5% milk in PBS-Tween20 0.1% (=PBS-T) to saturate the membranes. Ag-Fc fusion proteins were then detected in parallel through the Fc-portion, as well as their corresponding antigen to confirm, the formation of the fusion proteins. For that purpose, the Fc-portions were detected with an anti-mouse IgG coupled to Horseradish Peroxidase (HRP) (Dianova, Hamburg, Germany) while the membranes were stained in parallel with huIgG1 2G12 (Polymun Scientific, Klosterneuburg, Austria) or huIgG1 RGN10933 (18) antibodies followed by an incubation with an anti-human IgG coupled to HRP (Dianova, Hamburg, Germany) to detect gp120 or RBD, respectively. Subsequent to washes with PBS-T, target proteins were finally detected by the addition of Enhanced Chemiluminescence (ECL) reagent and pictures were captured by the Intas advanced fluorescence imager (Intas Science Imaging, Göttingen, Germany).

### Fc-fusion protein binding assay by flow cytometry

Functional activity of the Fc-fusion proteins was assessed by flow cytometry. HEK293T/17 cells stably expressing hAce2 and TZMbl cells expressing hCD4 were incubated for 30min at RT with serial dilutions of the cell supernatants containing the Ag-Fc fusion proteins. After 3 washing steps with FACS buffer (PBS + 2% FCS + 2mM NaN_3_), the cells were incubated with an anti-mouse IgG coupled to Cy-5 (Southern Biotechnology, Birmingham, USA, #SBA-1030-15) for 20min at RT followed by 3 washes with FACS buffer. Finally, cells were fixed with 2% paraformaldehyde (PFA) (Morphisto, Frankfurt am Main, Germany) for 20min at RT.

Binding inhibition assays were conducted by mixing the cell supernatants containing either the gp120 – Fc or the RBD – Fc variants with 20µg/mL of either VRC01 or RGN10933 antibodies. After a 1h incubation at 37°C, hAce2 expressing cells or TZMbl cells were stained with the antibody-cell SN mixtures as previously described above.

After fixation, samples were acquired onto an Attune Nxt cytometer (Thermo Fisher Scientific, Waltham, MA, USA) and analyzed using FlowJo V10 (FlowJo, LLC, Ashland, OR, USA).

### Purification of Ag-Fc fusion proteins

Briefly, Ag-Fc fusion proteins were produced in HEK293T/17 cells in D0 medium and purified 72h post-transfection by protein G chromatography using the Protein G Gravitrap™ columns (Cytiva, Marlborough, MA, USA) following the manufacturer’s recommendations. In short, Protein G Gravitrap™ columns were equilibrated with a two column volume of binding buffer (20mM sodium phosphate, pH 7.0) followed by the loading of the cell supernatants containing the Fc-fusion proteins. The loaded samples were then washed with a two column volume of binding buffer and the Ag-Fc proteins were eluted by the addition of 15mL of elution buffer (0.1M glycine-HCl, pH 2.7). The pH of the eluted fraction was directly neutralized by placing 1.5mL of 1M Tris-HCl, pH 9.0 at the bottom of the collection tube. After purification, the Fc-fusion proteins were buffer exchanged for PBS, pH 7.4 and concentrated using Pierce™ Protein Concentrators PES, 10K MWCO (Thermo Fisher Scientific, Waltham, MA, USA). After measuring the protein concentrations at A_280nm_ with a nanodrop, the samples were aliquoted and stored at -20°C.

### Fc-gamma receptors binding assay by ELISA

Prior to FcγRs binding assay, 10µg of each purified Fc-fusion protein were biotinylated using the One-step Antibody Biotinylation Kit (Milteny Biotec, Bergisch Gladbach, Germany) for 24h at RT. For performing the assay, 10ng per well of the recombinant murine Fc-gamma RI, RIIb, RIII (all R&D Systems, Minneapolis, MN, USA) or RIV (Abeomics, San Diego, CA, USA) were coated overnight at 4°C onto a high-binding 96-well plate in 100µL PBS. The next day, each well was blocked with 100µL of 5% milk in PBS-T for 1h at RT. Following three washes with 200µL PBS-T, 4-fold-serial dilution starting at 10µg/mL of the biotinylated Fc-fusion proteins were transferred to the plate and incubated for 2h at 37°C and then washed as described above. Each condition was incubated for 1h at RT with 100µL of a 1:250 dilution of Streptavidin-HRP (Thermo Fisher Scientific, Waltham, MA, USA). Subsequent to 3 washes with PBS-T, binding of the Fc-fusion proteins to the respective FcγRs was detected by the addition of 100µL of ECL per well and chemiluminescence, expressed as Relative Light Units (RLUs), was measured by a multilabel plate reader Victor X4 (Perkin Elmer, Hamburg, Germany).

### Animals housing and ethic statements

Female BALB/c mice, aged 5-6 weeks, were obtained from Charles River Laboratories (Wilmington, NC, USA) and held in ventilated cages at the Faculty of Medicine (Erlangen, Germany). All experimental procedures were approved under the license number 55.2-2532-2-1444 by the governmental authorities of Lower-Franconia.

### DNA immunization

DNA plasmids encoding for the respective immunogens were purified under endotoxin-free conditions and formulated at 500ng/µL in sterile PBS. Groups of twelve mice each were immunized by intramuscular (*i.m*.) DNA electroporation with the respective Ag-Fc encoding plasmids or an empty vector for the controls groups. Employing a 2.5mm 4-electrode array, BALB/c mice were injected with 20µg DNA in each *m. gastrocnemius* muscle (40µg total per animal) followed by electric pulses of 63V for 40ms with the TriGrid electrode array (Ichor Medical, San Diego, CA, USA). The animals were immunized twice with the same procedure with a three weeks interval between both immunizations. The control groups were housed and treated in the same manner as the experimental groups and therefore also represent adequate controls for the determination of age-dependent background levels in the antibody ELISAs.

### Analysis of the antigen-specific antibody response

All along the immunization experiments, blood samples were collected from the immunized mice by retrobulbar venous plexus puncture in order to test for the serum antigen-specific antibody levels. Blood samples were collected into Microtainer SST Tube (Becton Dickinson, Franklin Lakes, NJ, USA) and sera were isolated by 2min centrifugation at 11,000xg and transferred to new tubes. Additionally, the sera were heat-inactivated at 56°C for 30min and finally stored at -20°C for further use.

Antigen-specific IgGs were then evaluated by ELISAs. For that purpose, 100ng of either ConB gp120-His or SARS-CoV-2 delta variant RBD (ProteoGenix, Schiltigheim, France) were coated per well in high-binding 96 well-plates in 100µL PBS and stored overnight at 4°C. The day after, wells were blocked with 100µL of 5% milk in PBS-T for 1h at RT. Following 3 washes with 200µL of PBS-T, 100µL of the respective sera previously diluted at 1:400 for gp120 or 1:800 for RBD immunized groups into 2% milk in PBS-T were added to the plate and incubated for 1h at RT. After 3 washes, 100µL of polyclonal anti-mouse IgG antibodies coupled to HRP (Dianova, Hamburg, Germany) in 2% milk were transferred to each well and incubated for 1h at RT in the dark followed by 3 more washing steps. Finally, the total IgG levels were detected by the addition of 100µL ECL per well and chemiluminescence expressed in RLUs was captured with the multilabel plate reader Victor X4 (Perkin Elmer, Hamburg, Germany). The dynamic range of the chemiluminescence read-out spanned approximately 10,000 to 5,000,000 RLUs.

To determine serum concentrations of antigen-specific IgG subtpes, the same procedure was carried out against standards composed of the CD4bs b12 or the RGN10933 bearing the IgG1, IgG2a, IgG2b or IgG3 murine constant heavy chains and murine kappa constant light chain. Antibodies binding to the antigen were then detected with anti-mouse IgG1, IgG2a, IgG2b or IgG3 coupled to HRP (all from Southern Biotech, Birmingham, AL, USA), respectively.

Antigen-specific IgA levels were determined by coating 100ng of either ConB gp120-His or SARS-CoV-2 delta variant RBD in PBS and incubated overnight at 4°C. Plates were blocked for 1h at RT with 5% milk in PBS-T, washed 3 times with PBS-T and incubated for 1h at RT with 100µL of the mice sera diluted 1:200 into 2% milk in PBS-T. After three washes with PBS-T, 100µL of polyclonal anti-mouse IgA antibodies coupled to HRP (Thermo Fisher Scientific, Waltham, MA, USA) in 2% milk were transferred to each well and incubated for 1h at RT in the dark. IgA levels were then detected by the addition of ECL as described above.

The cut-off value for each ELISA was calculated as follows:


Cut−off= Mean (Empty vector group)+2×Standard Deviation (Empty vector group)

### Analysis of T helper cell response

Antigen specific T helper (Th) cell responses were analyzed in parallel by Intracellular Cytokine Staining (ICS) and quantitative IL-4 and IL-5 ELISA after restimulation of the splenocytes with antigen-specific peptides. To that end, spleens were obtained from immunized BALB/c mice *post-mortem* (week 5) and single cell splenocyte suspensions were prepared by mashing the spleens through Falcon® 70µm Nylon cell strainer (Somerville, Massachusetts, USA). Erythrocytes were lysed with ACK lysis buffer (0.15M NH_4_Cl; 0.01M KHCO_3_; 0.1mM Disodium EDTA; pH7.2 in H_2_O) for 8min at RT and the reaction was quenched by the addition of RPMI 1640 (Gibco, Thermo Fisher Scientific, Waltham, MA, USA) supplemented with 10% FCS, 1% Pen/strep, 2mmol L-glutamine and 10mmol HEPES (= complete RPMI medium).

The ICSs were conducted by stimulating 10^6^ splenocytes per condition in complete RPMI medium with 10µg/mL of an HIV Env MHC-II-restricted peptide GVPVWKEATTTLFCASDAKA (23) for ConB gp120 immunized mice, or with 2µg/mL of PepMix™ SARS-CoV-2 RBD peptide pool (#PM-SARS2-RBDMUT06-2, JPT Peptide Technologies, Germany) covering the delta variant RBD sequence for the RBD immunized groups. In both cases, the stimulation reactions were also composed of 2µg/mL anti-mouse CD28 (37.51; eBioscience, Frankfurt am Main, Germany) and 3µg/mL Brefaldin A (eBioscience, Frankfurt am Main, Germany) and cells were stimulated for 6h in a humidified incubator at 37°C. To detect antigen-specific CD4 T-cells, the stimulated splenocytes were surface-stained with anti-mouse CD4 coupled to SB600 (RM4-5, Biolegend, San Diego, CA, USA), anti-mouse CD8 FITC (53-6.7, East Rutherford, NJ, USA) and Fixable Viability Dye eFluor450 (eBioscience, Frankfurt am Main, Germany). After fixation with 2% PFA, the cells were permeabilized with 0.5% Saponin and stained intracellularly with anti-mouse TNFα Pe-Cy7 (MP6-XT22), anti-mouse IL-2 coupled to APC (JES6-5H4) and PE-conjugated anti-mouse IFNγ (XMG1.2) antibodies (all from eBioscience). The samples were acquired on an Attune Nxt flow cytometer and data were analyzed with FlowJo software. Gating strategy for the analysis of IFNγ+; TNFα+ and IL-2+ CD4+ T-cells is shown in **Supplementary Figure S2**.

In parallel, 10^6^ splenocytes per condition were also stimulated with the peptides described above and 2µg/mL of an anti-mouse CD28 antibody for 48h in the CO_2_ incubator. The cell supernatants were then collected, and IL-4, IL-5 and IL-10 concentrations were determined by quantitative ELISA using the IL-4, IL-5 and IL- Mouse Uncoated ELISA Kits (LifeTechnologies by Thermo Fisher Scientific, Waltham, MA, USA), according to the manufacturer instructions. The cut-off value for each assay was calculated as follows:


Cut−off= Mean (Empty vector group)+2×Standard Deviation (Empty vector group)

### SARS-CoV-2 pseudovirus neutralization assay

Neutralizing antibody activities in the sera of SARS-CoV-2 RBD immunized mice were evaluated by spike-pseudotyped Simian Immunodeficiency Virus particles as described previously (24). In brief, pseudotyped particles were produced in HEK293T/17 cells by co-transfecting vector DNA encoding for the respective SARS-CoV-2 spike, the SIV-based packaging vector and the luciferase transfer plasmid. The cell supernatants containing the pseudoviruses were collected 72h post-transfection, spun down for 5min at 2000xg to pellet cell debris, 0.45µm sterile-filtered and stored aliquoted at -20°C until further use.

One day prior to transduction, 2x10^4^ HEK293T/17 cells stably expressing hAce2 (25) were plated per well in complete DMEM medium into Flat-bottom 96-well plates. The day after, 10µL of each serum from RBD immunized mice were pooled for each group and 3-fold serially diluted in a final volume of 60µL in DMEM+1.5% FCS starting at a dilution factor of 20. Subsequently, the diluted sera were incubated for 1h at 37°C with 60µL of the respective SARS-CoV-2 pseudotypes, and 100µL of each condition were then transferred to the previously seeded HEK293T/17 cells expressing hAce2. After a 48h incubation in a humidified CO_2_ incubator, the cells were washed twice with 200µL PBS and the luciferase activity was detected by the addition of Bright-Glo™ lysis buffer (Promega) and measured using a luminometer (Berthold Orion II). Non-treated cells, as well as virus only conditions, were included in each assay and the basal luciferase activity of the cells were subtracted to each value using the non-treated cell condition. The percent of virus activity was then calculated as followed:


$$Percent\;of\;virus\;activity=\frac{RLU\;\left(tested\;pooled\;serum\;dilution\right)}{RLU\;\left(virus\;only\;condition\right)}\times 100$$

The Inhibitory Dose 50% (ID50) corresponding to the dilution factor of the sera that reduced the virus activity to 50% was then determined by GraphPad Prism. Each assay was conducted at least twice including duplicates of the pooled sera dilutions.

### Statistical analyses

Statistical analyses were performed with GraphPad Prism software 6 (Graphpad Software Inc., San Diego, CA, USA) and significant differences were determined by One-way ANOVA followed by Tukey’s multiple comparison test or with Kruskal-Wallis test with Dunn’s post test as indicated in the figure legends. Significance between each group was considered at *p*<0.05.

## Results

### Design of DNA immunogens encoding Fc-fusion proteins

**Figure 1:**
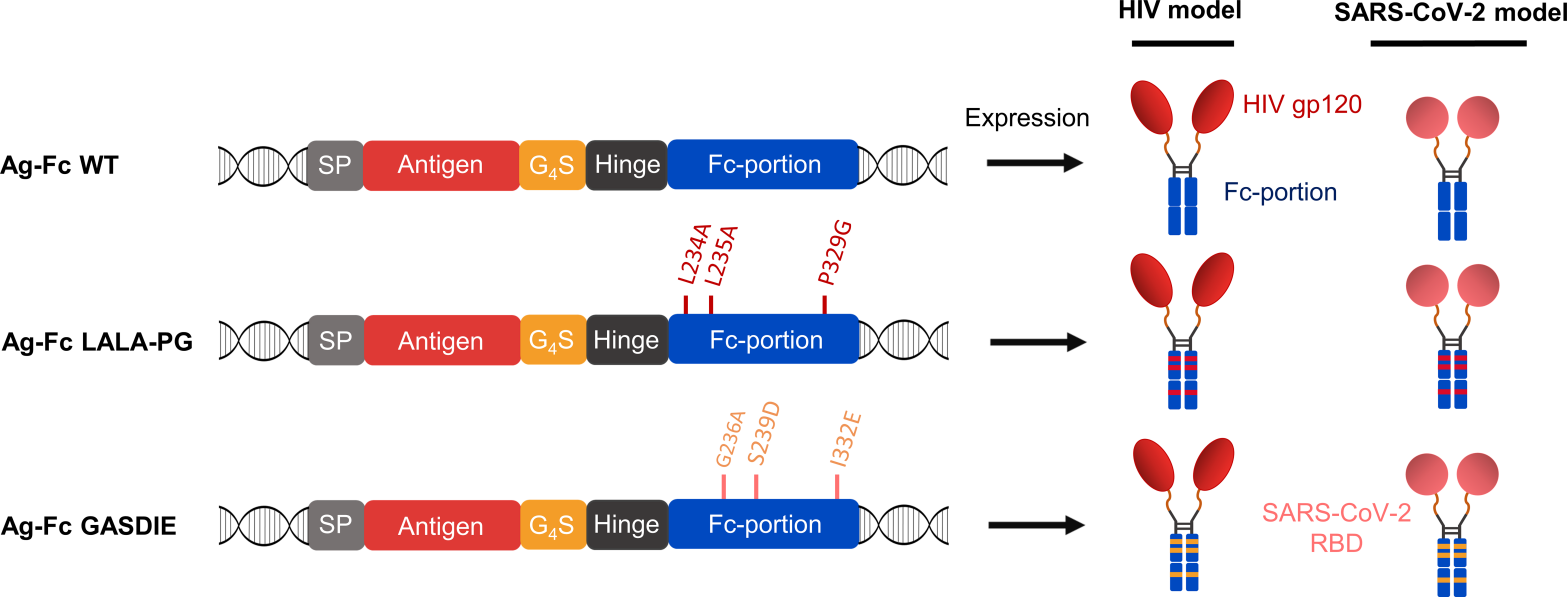
Antigen design. Schematic diagrams of the expression cassette for the fusion proteins (left panel). The antigens of interest were fused downstream of the mouse Ig heavy chain signal peptide (=SP) MGWSCIILFLVATATGVQC via a Gly4Ser linker (= G_4_S) to a murine IgG2a Fc fragment with the mutations affecting binding to FcγRs indicated. HIV gp120 and SARS-CoV-2 RBD were cloned as antigens of interest and the expected quaternary structures of the expressed fusion proteins are shown (right panel).

To evaluate the influence of Fc-effector functions on the immunogenicity of Fc-fusion proteins, we designed expression plasmids encoding viral antigens fused to the muIgG2a Fc fragment having the highest Fc-effector function abilities among all murine IgG subtypes (26, 27). Hypothesizing that immunization with Fc-fusion proteins leads to higher antibody titers by enhancing antigen uptake and/or antigen presentation through Fc-gamma receptors interaction on APCs, we sought to explore whether altering Fc-effector functions of the Fc-fusion proteins has an impact on the humoral immune response towards the viral antigens. Therefore, the L234A/L235A/P329G (LALA-PG) or G236A/S239D/I332E (GASDIE) described for impairing or improving Fc-gamma receptors binding (28–30), respectively, were introduced into the Fc-portion of the muIgG2a DNA coding sequence. Each construct consists of a signal peptide followed by the viral antigen of interest, linked by a Gly_4_Ser linker (G_4_S linker) to the muIgG2a hinge-Fc-portion sequence (**Figure 1**, left panel, **Figure S1**).

Since viral antigens can differ in the IgG subtype response, the study was conducted with two different viral antigens, namely gp120 of HIV-1 and the RBD of SARS-CoV-2. The HIV-1 gp120 was selected as antigen due to its unusual induction of a strong IgG1 dominated antibody response in mice, which was associated with the glycosylation of gp120 (31). In contrast, the IgG1 to IgG2a/c subtype response to the RBD is more balanced in mice (32, 33).

Of note, all antigens were cloned in the same pFuse expression plasmid. Overall, 6 different antigens were designed for this study (**Figure 1**, right panel).

### Characterization of Fc-fusion proteins

**Figure 2:**
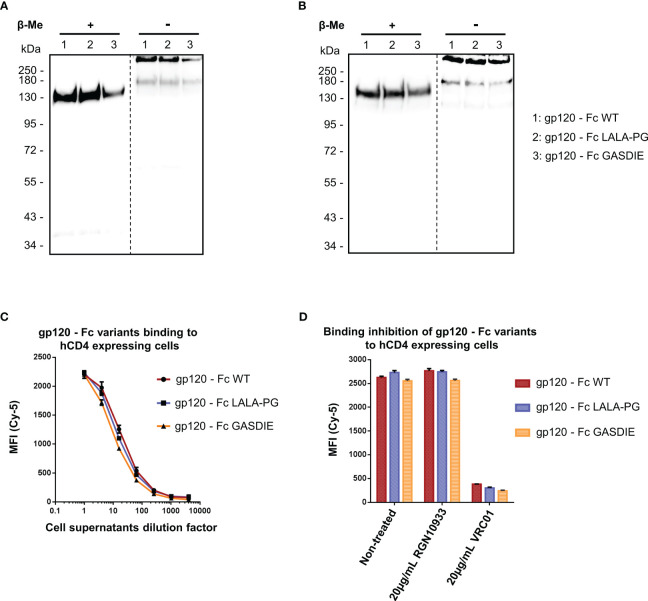
Expression and functional analysis of gp120 antigens. **(A, B)** Supernatants of cells transfected with the indicated gp120 – Fc fusion proteins were analyzed by western blots in presence or absence of the reducing agent β-Me. The Fc-fusion proteins were detected by **(A)** an anti-mouse IgG antibody coupled to HRP and **(B)** the 2G12 antibody, a human IgG1 to HIV-1 Env followed by an anti-human IgG antibody coupled to HRP. The dotted lines represent the truncation between the blot pictures **(C)** Four-fold serial dilutions of the cell supernatants containing the gp120 – Fc variants proteins were incubated with hCD4 expressing TZMbl cells. Binding of the Fc-fusion proteins to hCD4 expressing cells were detected via an anti-mouse IgG antibody coupled to Cy-5 and samples were analyzed by flow cytometry. **(D)** Prior to staining of TZMbl cells, the undiluted cell supernatants containing the Fc-fusion proteins were pre-incubated 1h at 37°C with either 20µg/mL of the CD4bs anti-HIV Env VRC01 antibody, 20µg/mL of the anti-RBD RGN10933 antibody or with DMEM medium (= Non-treated control). TZMbl cells were then stained as described above and binding inhibition was determined by flow cytometry.

To characterize the gp120 – Fc variants, the expression plasmids were transfected into HEK293T/17 cells and the supernatants were analyzed by western blot via both polyclonal anti-mouse IgG and anti-HIV Env 2G12 antibodies targeting the Fc-portion or the gp120 subunits of the fusion proteins, respectively. Under reducing conditions, a single 150kDa band corresponding to the monomer of the Fc-fusion variant was observed for the WT and the mutants by staining with the anti-mouse antibody. Staining with the HIV Env antibody revealed the expected predominant band at 150kDa and a weak band at 120 kDa, most likely representing a premature termination product of the fusion protein (**Figures 2A, B**). Under non-reducing conditions, a predominant form of the Fc-fusion proteins could be observed above 250kDa consistent with the dimerized chains of the Fc-fusion proteins. The minor products at 180kDa and 120kDa are potentially the result of incomplete formation of the Fc-fusion proteins or dissociation of the dimers during SDS gel electrophoresis, running higher due to the conserved tertiary structure in non-reducing conditions and represent a monomer chain and gp120 alone, respectively. Overall, the expression of all gp120 – Fc variants showed a predominant formation of the expected Fc-fusion proteins in the cell supernatants of HEK293T/17 cells with comparable expression levels as detected by western blot.

A functional binding assay using serial dilutions of cell supernatants containing the gp120 – Fc variant proteins was conducted on TZMbl cells expressing the human CD4 (hCD4) molecule by FACS analysis (**Figure 2C**). The detection using a fluorescently labelled anti-mouse IgG revealed dose-dependent binding of the gp120 – Fc fusion proteins to hCD4 expressing cells, while binding to non-CD4 expressing cells could not be detected (data not shown). In addition, binding to hCD4 expressing cells was inhibited by pre-incubating the cell supernatants containing the Fc-fusion proteins with 20µg/mL of the anti-HIV Env VRC01 antibody, but not with the anti-RBD RGN10933 antibody, confirming that binding of the gp120 – Fc fusion proteins to hCD4 was mediated by gp120 as expected (**Figure 2D**). Importantly, all gp120 – Fc variants displayed relatively similar magnitude and dose-dependent binding to TZMbl cells which also indicates comparable expression levels *in vitro*.

**Figure 3:**
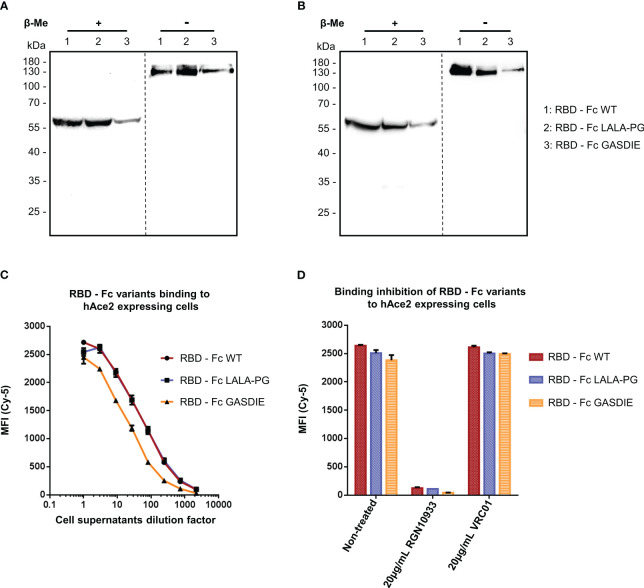
Expression and functional analysis of RBD antigens. **(A, B)** Cell supernatants of HEK293T/17 cells, transfected with the expression vectors encoding for the RBD – Fc fusion proteins were analyzed by western blot in the presence or absence of β-Me. The Fc-fusion proteins were detected by **(A)** an anti-mouse IgG antibody coupled to HRP or by **(B)** the anti-RBD huIgG1 RGN10933 antibody followed by an anti-human IgG coupled to HRP. The dotted lines correspond to the truncation of the blot pictures **(C)** Four-fold serial dilutions of the cell supernatants containing the RBD – Fc fusion proteins were incubated with hAce2 expressing cells. Binding of the Fc-fusion proteins to hAce2 expressing cells was detected by an anti-mouse IgG antibody coupled to Cy-5 and samples were analyzed by flow cytometry. **(D)** Prior to staining of hAce2 expressing cells, the undiluted cell supernatants were pre-incubated 1h at 37°C with either 20µg/mL of the CD4bs anti-HIV Env VRC01 antibody, 20µg/mL of RGN10933 antibody or with DMEM medium (= Non-Treated control). The cells were then stained as described above and binding inhibition was determined by flow cytometry.

In a similar manner, the expression of the RBD – Fc variants could be detected by western blot analysis with both polyclonal anti-mouse IgG antibodies and human anti-SARS-CoV-2 Spike RGN10933 antibody in the cell supernatants of HEK293T/17 cells. In both staining conditions, the monomeric form of the Fc-fusion proteins could be detected at 60kDa under reducing and ~130kDa in non-reducing conditions with no side products due to non-pairing of the Fc-portion (**Figures 3A, B**). However, expression levels of the RBD – Fc GASDIE variant appeared to be lower in the representative experiment shown and in repeat experiments.

RBD – Fc variants contained in the cell supernatants were characterized through binding assay on HEK293T/17 cells stably expressing hAce2 on the cell surface and detected via a monoclonal anti-mouse IgG. FACS analysis revealed a dose-dependent binding to hAce2 expressing cells in a dose-dependent manner (**Figure 3C**). Here, a similar binding magnitude of RBD – Fc WT and LALA-PG variants was observed, while RBD – Fc GASDIE showed weaker binding signal potentially due to lower expression levels *in vitro*. Importantly, binding of the Fc-fusion proteins to hAce2 expressing cells was inhibited for all RBD – Fc variants by a prior incubation with 20µg/mL of the anti-RBD RGN10933 antibody but not by the anti-HIV antibody VRC01 (**Figure 3D**). Altogether, these results indicate that the RBD – Fc variants were successfully dimerizing into Fc-fusion proteins with a functional RBD capable of binding hAce2 specifically.

To confirm the effect of the point mutations in the Fc fragment on binding of the fusion proteins to murine FcγRs, the latter were coated on ELISA plates. The gp120 – Fc and RBD – Fc variants were produced in HEK293T/17 cells and purified by Protein G chromatography from the cell supernatants. Calculation of the protein yield after purification confirmed a lower expression of RBD - Fc GASDIE (**Supplementary Table 1**). Coomassie Blue staining of SDS-Page gels revealed that the Fc-fusion proteins were successfully purified and displayed bands comparable in size to those previously seen in the western blot analyses of HEK293T/17 cell supernatants (**Figure S3**). Binding of serial dilutions of the purified and biotinylated Fc-fusion proteins to the FcγR-coated ELISA plates was then determined.

**Figure 4:**
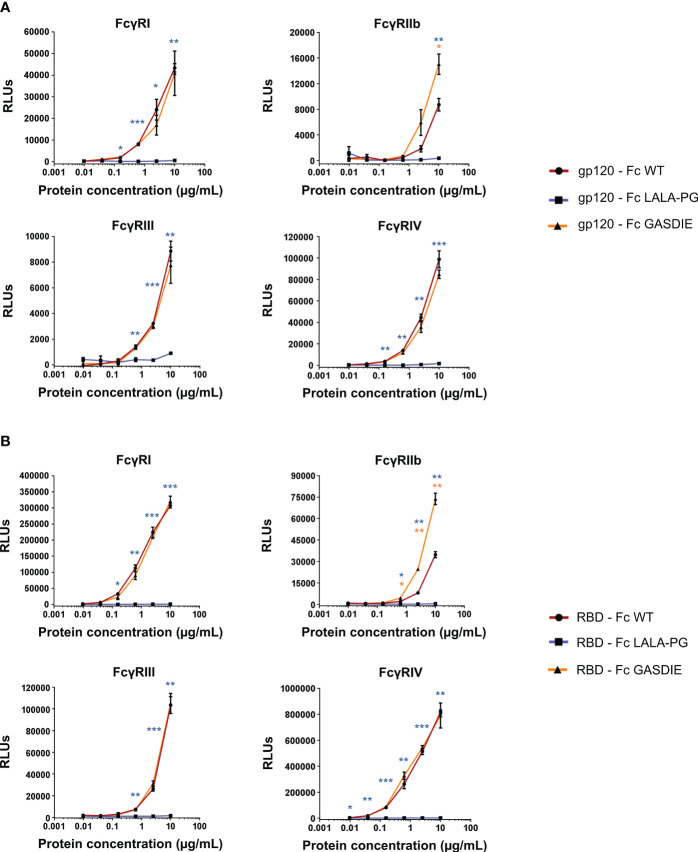
Binding of the Fc-fusion proteins to murine FcγRs. Four-fold serial dilutions of the purified and biotinylated gp120 – Fc **(A)** and RBD – Fc **(B)** fusion proteins were incubated with recombinant murine FcγRs in an ELISA-based assay. Binding of Fc-fusion proteins was detected by HRP coupled streptavidin. Mean of duplicates and SD are displayed on each graph. One representative out of two independent experiments is shown. The statistical significance of observed differences of gp120 – Fc WT and RBD – Fc WT to the mutants was calculated for each concentration by One-Way ANOVA followed by Tukey’s post test; *p*-*values* indicate significant differences (* *p<*0.05; ** *p<*0.005; *** *p<*0.0005).

As expected, the insertion of the LALA-PG mutation in the Fc-portion fused to both gp120 and RBD completely abrogated FcγRs binding compared to the non-mutated Fc-fusion proteins (**Figures 4A, B**). Interestingly, and although the GASDIE mutation enhanced FcγRIIb, III and IV binding once inserted into the anti-HIV Env b12 muIgG2a monoclonal antibody (**Figure S4**), it only resulted in a significant improved binding to FcγRIIb for both mutated viral antigen Fc-fusion proteins but showed similar binding activity than the respective non-mutated antigens for the other FcγRs. In summary, the insertion of the LALA-PG mutation in the Fc-portion greatly impaired the FcγR binding of the Ag-Fc fusion proteins designed for this study, while the GASDIE mutation only resulted in the improvement of binding to FcγRIIb *in vitro*.

To further characterize the impact of the Fc-mutations on activation of the complement system, mouse C1q binding assays were performed by ELISA using mouse complement serum. Consistently with the FcγRs binding impairment, the LALA-PG mutation significantly abrogated the binding of C1q over the non-mutated Fc-fusion proteins (**Figure S5**). However, the GASDIE Fc-fusion variants showed a moderate but significant reduced ability to activate the complement system compared to the WT fusion proteins.

### Immunogenicity of gp120 – Fc fusion proteins

**Figure 5:**
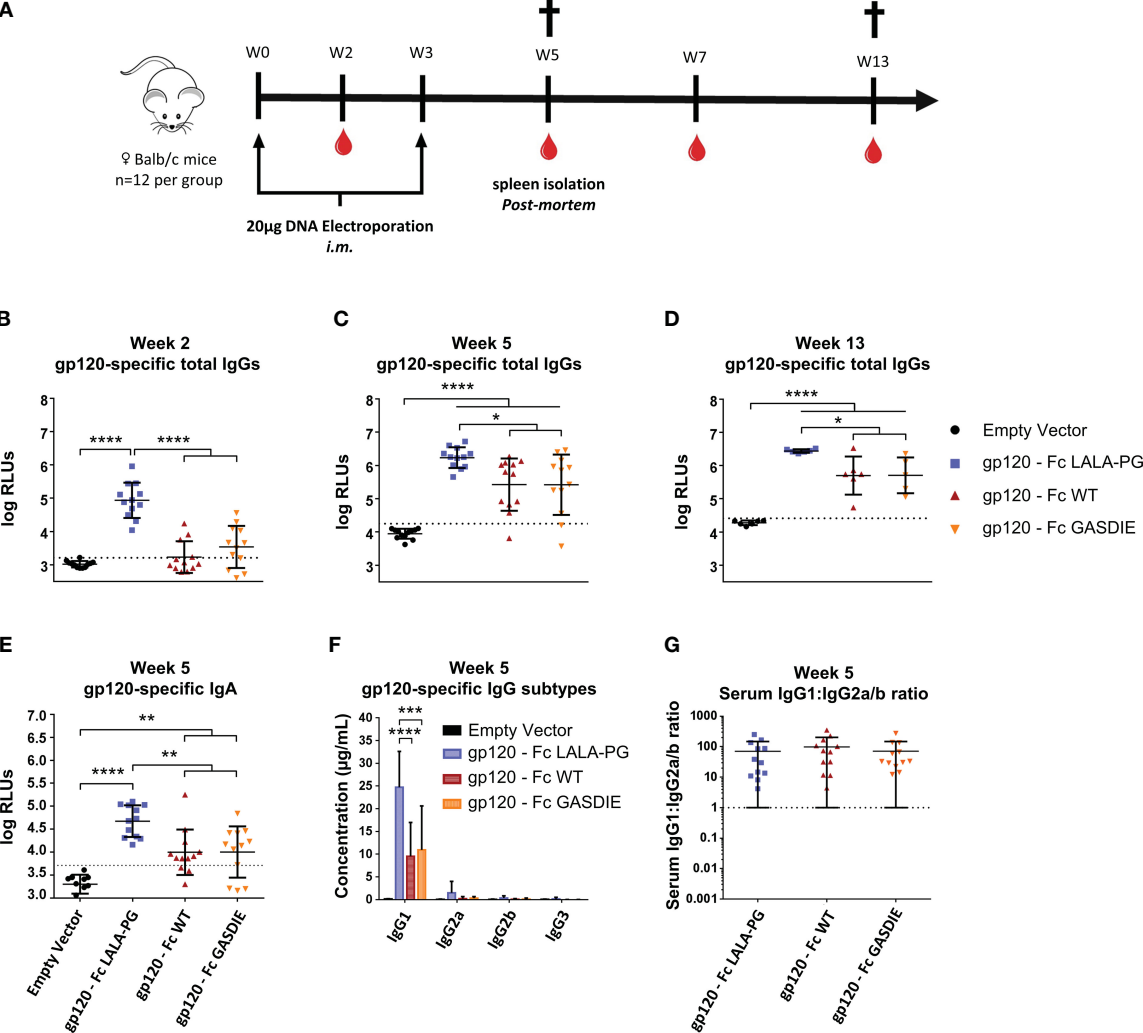
Immunogenicity of gp120 – Fc fusion proteins. **(A)** Immunization schedule. Twelve female BALB/c mice per group were immunized on week 0 (W0) and week 3 (W3) by intramuscular (*i.m*.) electroporation of plasmid DNAs encoding the Fc-fusion proteins indicated. Blood samples were collected throughout the experiment as indicated by the blood drop symbol and spleens from half of the animals were isolated at week 5 (W5). (**B–D**) Gp120-specifc IgG levels in the sera of the mice at weeks 2, 5 and 13 were determined by ELISA at a sera dilution of 1:400 and are expressed as Relative Light Units (RLUs). The cut-off values for each ELISA are represented by a dotted line. Bars represent mean and SD of the groups overlaid with individual data point (n=12 at weeks 2 and 5; n=6 at week 13). Significant differences between groups as determined by One-Way ANOVA followed by Tukey’s multiple comparison test are indicated (* p<0.05; ** p<0.005; *** p<0.0005; **** p<0.0001). **(E)** Gp120-specific IgA levels in the sera of the immunized mice at week 5 were determined by ELISA at the sera dilution of 1:200. The cut-off value is indicated by the dotted line. Significant differences between groups as determined by One-Way ANOVA followed by Tukey’s multiple comparison test are indicated (* p<0.05; ** p<0.005; *** p<0.0005; **** p<0.0001). **(F)** Serum concentration of gp120-specific IgG subtypes at week 5. Bars represent mean and SD of the groups. Significant differences between groups as determined by One-Way ANOVA followed by Tukey’s multiple comparison test are indicated (* p<0.05; ** p<0.005; *** p<0.0005; **** p<0.0001) **(G)** Ratio of IgG1 to IgG2a/b serum concentrations at week 5. Each bar represents the mean and SD of the respective group overlaid with the data points of individual mice. Significant differences between groups were determined by One-Way ANOVA followed by Tukey’s multiple comparison. No statistical significance was observed between the Fc-fusion variant groups (p<0.05).

To determine whether the inserted mutations into the Fc fragment modulate the immunogenicity of Fc-fusion proteins, female BALB/c mice were immunized twice in a three-week interval by intramuscular (*i.m.)* electroporation of 40 µg plasmid DNA encoding the respective gp120 antigens or an empty vector control (**Figure 5A**). Gp120-specific total IgGs were analyzed in the isolated sera of the animals throughout the immunization study up to 13 weeks after the first immunization. Surprisingly, and contrary to our initial hypothesis, the immunizations with gp120 – Fc WT and gp120 – Fc GASDIE elicited significantly lower anti-gp120 IgG levels than the LALA-PG mutated Fc-fusion construct (**Figure 5B**). As expected, the second DNA immunizations boosted IgG-specific antibody levels in all groups and gp120 – Fc WT and GASDIE variant immunized animals displayed significantly higher serum antibody levels than the mock-vaccinated control group (**Figures 5C, D**). Nonetheless, the gp120-specific IgG levels in the sera of the DNA immunized groups with gp120 – Fc LALA-PG remained significantly higher throughout the observation period of the experiment. Additionally, gp120 – Fc LALA-PG immunization led to the induction of significantly higher gp120-specific IgA levels in the sera of the immunized mice compared to the Fc-effector function competent Fc-fusion variants (**Figure 5E**).

Since certain antibody subtypes, in particular IgG2a in the murine model, retain higher Fc-effector functions and correlate with better protection against infection and greater maintenance of HIV viremia (34–36) we also explored whether the mutations in the Fc fragments of the fusion proteins also affect the IgG subtype response. For that purpose, antigen-specific antibodies in the sera of immunized BALB/c mice were quantified by HRP-conjugated subtype-specific secondary antibodies in ELISAs using the anti-HIV Env b12 Fab-fragment fused to the murine Fc fragments of the IgG heavy chain subtypes as standards. The DNA immunizations encoding the gp120 fusion proteins elicited a dominant antigen-specific IgG1 response. As for the total IgG levels, the LALA-PG mutant of the fusion protein induced higher IgG1 antibody responses than the WT and GASDIE fusion proteins (**Figure 5F**). A similar trend was seen for the other IgG subtypes, although the difference did not reach statistical significance. Thus, the ratio of gp120-specific IgG1 to IgG2a+IgG2b (=IgG1:IgG2a/b ratio) serum concentrations was not affected by the Fc mutations (**Figure 5G**).

**Figure 6:**
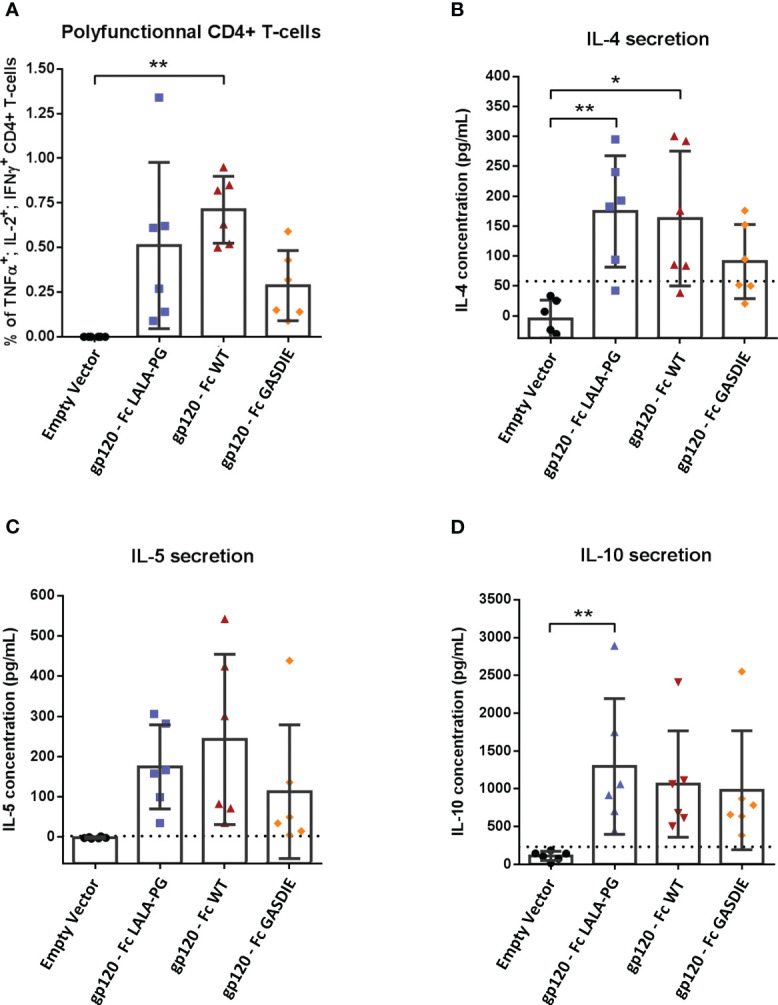
Gp120-specific T-helper cell responses to gp120 – Fc fusions proteins. **(A)** Splenocytes from immunized mice at week 5 were isolated and stimulated for 6h with an HIV Env MHC-II restricted peptide in presence of Brefaldin A and a CD28 antibody. CD4+ T-cells expressing TNFα, IL-2 and IFNγ were analyzed by FACS following an intracellular cytokine staining. **(B–D)** Splenocytes were stimulated with an HIV Env MHC-II restricted peptide in presence of CD28 antibody for 48h. Cell supernatants were harvested, diluted 1:3 and IL-4 **(B)**, IL-5 **(C)** and IL-10 **(D)** concentrations were determined by quantitative ELISAs. The cut-off value is indicated by the dotted line on each graph. Bars represent mean and SD of each group overlaid with individual data points (n=6 per group). Significant differences as determined by One-Way ANOVA followed by Tukey’s multiple comparison test are indicated (* *p<*0.05; ** *p<*0.005).

To evaluate whether Fc-effector functions of the fusion proteins affect T-helper (Th) cell type responses and if potential differences correlate with antibody responses, spleens from immunized mice (n=6 per group) were collected after the second immunization. The Th1- and Th2-associated cytokines were evaluated by Intracellular Cytokine Staining (ICS) and quantitative ELISA after restimulation of the splenocytes with an immunodominant gp120 peptide described previously (23). All groups immunized with the gp120 fusion proteins displayed detectable elicitation of Th-1-associated IFNγ, TNFα and IL-2 CD4+ T-cells compared to the mock immunized control group, although no statistical difference for the elicitation of polyfunctional CD4+ T-cells was observed among the gp120 immunized animals (**Figure 6A**). In line with the results from the Th1 cells, similar outcomes were observed for IL-4 and IL-5 secretion (**Figures 6B, C**). Immunizations with the different gp120 immunogens also elicited comparable secretion of the immunosuppressive IL-10 cytokine (**Figure 6D**).

### Immunogenicity of the RBD – Fc fusion proteins

**Figure 7:**
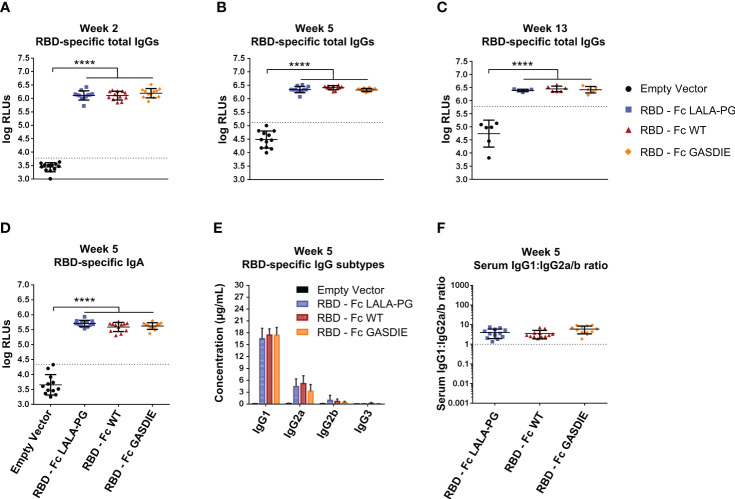
Immunogenicity of RBD – Fc fusion proteins. **(A–C)** RBD-specific IgG levels in the sera of the mice on weeks 2, 5 and 13 were determined by ELISA at a sera dilution of 1:800 and expressed as Relative Light Units (RLUs). The cut-off values for each ELISA are represented by a dotted line. Bars represent mean and SD of the groups overlaid with individual data point (n=12 at weeks 2 and 5; n=6 at week 13). Significant differences between groups as determined by One-Way ANOVA followed by Tukey’s multiple comparison test are indicated (**** p<0.0001). **(D)** RBD-specific IgA levels in the sera of the immunized mice at week 5 were determined by ELISA at the sera dilution of 1:200. The cut-off value is indicated by the dotted line. Significant differences between groups as determined by One-Way ANOVA followed by Tukey’s multiple comparison test are indicated (**** p<0.0001). **(E)** Serum concentration of RBD-specific IgG subtypes at week 5. Bars represent mean and SD of the groups. Significant differences between groups as determined by One-Way ANOVA followed by Tukey’s multiple comparison test are indicated No statistical significance was observed between the Fc-fusion variant groups (p<0.05). **(F)** Ratio of IgG1 to IgG2a/b serum concentrations at week 5. Each bar represents the mean and SD of the respective group overlaid with the data points of individual mice. Significant differences between groups were determined by One-Way ANOVA followed by Tukey’s multiple comparison. No statistical significance was observed between the Fc-fusion variant groups (p<0.05).

To further investigate whether immunogenicity of Fc-fusion proteins is dependent on the fused viral antigen, we conducted similar immunization studies with the RBD of SARS-CoV-2. For that purpose, 6-8 weeks-old female BALB/c mice were DNA electroporated *i.m.* twice with a 3 weeks interval with the RBD antigens (**Figure 5A**). RBD-specific IgG levels in the sera of immunized mice were assessed regularly throughout the immunization study. The first DNA immunization with all RBD antigens elicited significant RBD-specific IgG responses in comparison to the mock immunized group (**Figure 7A**). As expected, a second immunization with the respective immunogens boosted the antigen-specific total IgG response. However, no significant difference was observed between the RBD – Fc variants immunized groups all along the study (**Figures 7B, C**). In line with the RBD-specific IgG levels, antigen-specific IgA could be detected in the sera of each immunized animal while no statistical difference was observed among the different immunized groups (**Figure 5D**).

Regarding RBD-specific antibody subtypes, the immunization with the RBD – Fc fusion variant expression vectors induced a dominated IgG1 response with detectable anti-RBD IgG2a and IgG2b, while no statistical difference was observed between the immunized groups (**Figure 7E**). Of note, no RBD-specific IgG3 elicitation could be detected in the sera of the immunized mice. Overall, the antigen-specific IgG1:IgG2a/b serum concentration ratio remained similar among the RBD immunized groups and displayed a more balanced IgG1:IgG2a/b antibody response than seen with the gp120 immunizations (**Figure 7F**).

**Figure 8:**
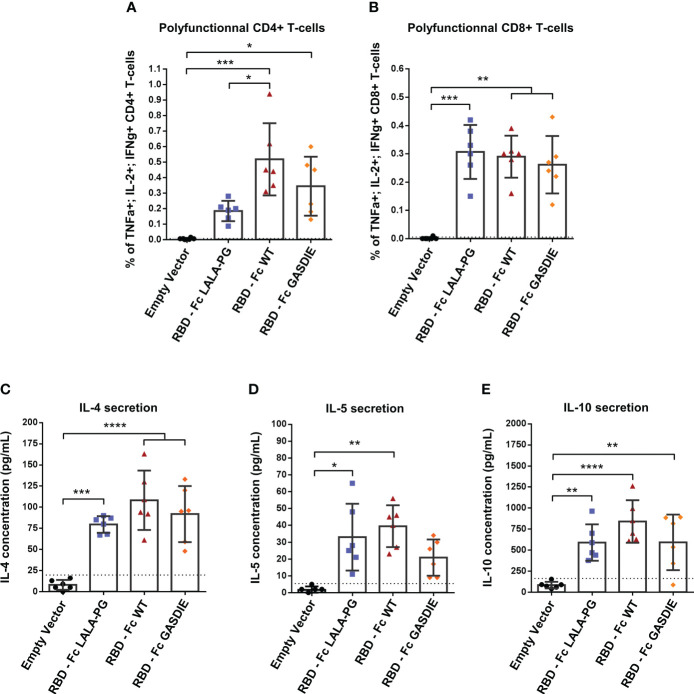
RBD-specific CD4 and CD8 T cell responses after immunization with RBD – Fc fusion proteins. Splenocytes from immunized mice at week 5 were isolated and stimulated for 6h with a RBD peptide pool in presence of Brefaldin A and a CD28 antibody. CD4+ **(A)** and CD8+ **(B)** T-cells expressing TNFα, IL-2 and IFNγ were analyzed by FACS following an intracellular cytokines staining. **(C, D)** Splenocytes were stimulated with a RBD peptide pool in presence of CD28 antibody for 48h. Cell supernatants were harvested, diluted 1:3 and IL-4 **(C)**, IL-5 **(D)** and IL-10 **(E)** concentrations were determined by quantitative ELISAs. The cut-off value is indicated by the dotted line. Bars represent mean and SD of each group overlaid with individual data point (n=6 per group). Significant differences as determined by One-Way ANOVA followed by Tukey’s multiple comparison test are indicated (* *p<*0.05; ** *p<*0.005; *** *p*<0.005; **** *p*<0.0001).

To assess the T-helper cell responses induced by the RBD immunogens, the splenocytes of immunized BALB/c mice were stimulated with 2µg/mL of a peptide pool covering the SARS-CoV-2 delta variant RBD amino acid sequence. Th1-assiociated cytokines expression were assessed by ICS among CD4+ T-cells as well as CD8+ T-cells since the peptide pool could stimulate both antigen-specific T-cell subsets. As expected, a strong induction of CD4+ Th1 cells were elicited by all RBD immunizations although RBD – Fc LALA-PG led to significantly lower CD4+ Th1-associated cellular responses than the other immunogens (**Figure 8A**). Besides, all RBD immunizations induced similar levels of antigen-specific CD8 T-cells as observed by intracellular expression of IFNγ+, TNFα+ and IL-2+ among CD8+ T-cells (**Figure 8B**). In addition, no difference between the RBD immunized groups was observed for the secretion of IL-4, IL-5 and IL-10 into the cell supernatants of restimulated splenocytes (**Figures 8C–E**).

**Figure 9:**
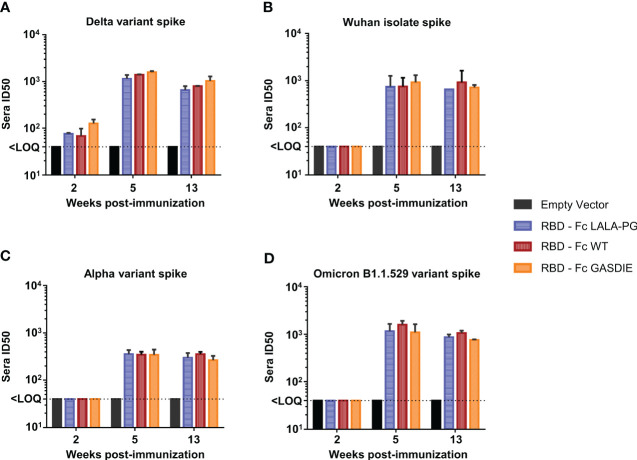
Neutralizing antibody titers against SARS-CoV-2 spike variants after immunization with RBD – Fc fusion proteins. Sera from each group of immunized mice were pooled and tested in a pseudovirus neutralization assays against the spike proteins of the SARS-CoV-2 delta variant **(A)**, the Wuhan isolate **(B)**, the Alpha variant **(C)** and Omicron B1.1.529 variant **(D)**. Pooled sera were serially diluted starting at 1:20, incubated for 1h at 37°C with the corresponding pseudoviruses and transferred to hAce2 expressing HEK293T/17 cells. Bars represent the mean and SDs of the 50% inhibitory dilution (ID_50_) of each group from two independent experiments. The Limit of Quantification (LOQ) represents the minimum dilution factor used for the assays and is represented by the dotted line. Significant differences as determined by One-Way ANOVA followed by Tukey’s multiple comparison test for each time point are indicated. No statistical significance was observed between the Fc-fusion variant groups (p<0.05).

To explore whether anti-RBD IgG levels correlated with neutralization activities against SARS-CoV-2, neutralization assays using pseudotyped particles with the SARS-CoV-2 delta variant spike were conducted by pooling the sera of each group of immunized mice. Significant neutralization activity could be detected against the delta variant after prime-immunization (week 2) although no difference was observed among the immunized groups (**Figure 9A**). After the second immunization, all immunized groups displayed more potent neutralizing activity correlating with the increase of anti-RBD IgG levels. Immunizations with the RBD – Fc variants led to comparable neutralizing antibody activities in the sera of the different immunized groups. To further investigate whether the Fc-effector functions could induce higher somatic hypermutations in B-cell lineages, and therefore provide a better cross-neutralization against SARS-CoV-2 variants, the neutralization assays were extended to SIV vectors pseudotyped with the SARS-CoV-2 Wuhan isolate spike, the alpha variant spike, and the Omicron B1.1.529 variant spike (**Figures 9B–D**). Here, neutralizing activities against the tested variants were only detectable after boost-immunization with the respective constructs. In line with the anti-RBD specific IgG levels detected in the sera of mice immunized with the RBD – Fc variants, no statistical difference was observed among these groups of animals.

Taken together, the RBD – Fc variant immunizations led to comparable antigen-specific serum IgG concentrations displaying similar neutralizing activities against the SARS-CoV-2 delta variant as well as the Wuhan isolate, Alpha and Omicron B1.1.521 variants. Additionally, all RBD immunogens led to the induction of similar levels of antigen-specific CD8+ and Th2 CD4+ T-cells, while immunization with RBD – Fc LALA-PG led to a significantly lower induction of Th1 cells which did not correlate with differences in IgG levels. Therefore, we conclude that Fc-effector functions did not modulate the immune responses towards the RBD.

In summary, and although the inserted mutations into the Fc-fusion proteins retained similar Fc-effector function properties independently of the fused antigen as determined *in vitro* (**Figures 4A, B** **,** **S5**), the Fc-effector functions conferred to gp120 led to a lower immunogenicity towards the antigen while the humoral immune response towards the RBD was not influenced by Fc-effector mechanisms.

## Discussion

To explore the influence of FcγR binding of Fc-fusion proteins on their immunogenicity, we constructed six expression plasmids for Fc-fusion proteins: HIVgp120 and the RBD of SARS-CoV-2 were each fused to WT Fc, a LALA-PG mutant of Fc and a Fc GASDIE mutant. Functional assays with gp120 and RBD – Fc variants demonstrated the proper design of the immunogens as shown by binding to hCD4 and hAce2 expressing cells, respectively. In accordance with previous studies, the LALA-PG mutated Fc-fusion proteins led to the abrogation of FcγR binding activity for both antigen Fc fusion proteins analyzed. The GASDIE mutation only improved FcγRIIb over the non-mutated Fc-portion. Of note, the GASDIE mutation was previously described to improve Fc-effector functions of human IgG1 but, to the best of our knowledge, not for the murine IgG2a. Here, our results indicate that the insertion of the GASDIE mutation in a recombinant muIgG2a antibody improved FcγRIIb, III and IV binding (**Figure S4**), but only enhanced FcγRIIb binding in the context of Fc-fusion proteins (**Figure 4**).

DNA immunization of BALB/c mice revealed that the FcγR-binding gp120 – Fc variants induced lower gp120-specific IgG and serum IgA levels compared to the LALA-PG fusion protein deficient for FcγR-binding and complement system activation. Conversely, the RBD immunogens induced similar outcomes in terms of humoral immune response, independently of their Fc-effector function abilities. Hence, our study highlights that the outcome for Fc-fusion protein immunization relies not only on the Fc-effector functions conferred by the Fc-portion but can depend on the fused antigen.

Why FcγR-binding of the fusion protein reduced the antibody to HIV Env, but not RBD is unknown. We observed previously that the HIV Env protein induces an unusual IgG1 dominated antibody response in mice, while the antibody response to HA of influenza A virus and the F protein of RSV is more balanced (31). Mechanistically, evidence for a role of the glycosylation of HIV Env was obtained (31). Dysregulation of dendritic cell (DCs) maturation and functionalities upon gp120 interactions have been repeatedly demonstrated (37–39). Although we did not find direct evidence, it is plausible that cross-linking FcγRs with C-type Lectin receptors on APCs could accentuate the dysregulation of DCs and therefore lead to a reduced induction of anti-gp120 antibodies. It is also conceivable that Fc-effector functions shift the uptake of the gp120 - Fc fusion protein from C-type lectin on APCs to a different subset of APCs via FcγRs interactions which are less efficient at processing and/or presenting the antigen to the adaptive immune system. The absence of detectable differences in our analyses of T helper cell responses argues against this hypothesis, but T follicular helper cell profiles and Th17 and regulatory T cell responses remain to be evaluated.

Our results differ from the findings of Chen et al. (40). This study reported higher HIV-1 gp120 antibody levels in one pool of sera from three mice immunized with gp120 fused to wild type murine IgG2a than in one pool of sera from three mice immunized with a L234V-L235A mutant of the same gp120 Fc fusion protein (40). Due to the small number of animals analyzed only as one pool per group, statistical analyses were not possible. In addition, different modes of delivery of the Fc-fusion proteins were used in our study and the study by Chen et al. that may also affect both the immunogenicity of the constructs and the influence of the FcγR binding activity of the HIV Env Fc fusion proteins. Furthermore, the mice strain used for the immunization study conducted by Chen et al. has not been mentioned and could therefore account for the divergent results of our study. Last but not least, the recombinant proteins used in the study by Chen et al. were produced in insect cells which may lead to a different glycosylation pattern of recombinant proteins compared to those produced in mammalian cells. In a rhesus monkey experiment, CpG adjuvanted HIV Env gp120 fused to the Fc fragment of rhesus monkey IgG1 led to higher antibody responses than HIV Env gp120 not fused to Fc (9). Since no mutants with impaired in FcγR binding were included, dimerization and/or a prolonged half-life may also explain the improved antibody responses observed.

Our observation of immunoinhibitory effects of the Fc fragment is in line with other studies, demonstrating that Fc-fusion proteins can have an adverse influence on the induction antigen-specific IgG responses. For instance, immunization of BALB/c mice with MERS-CoV spike protein fused to human IgG4 Fc-portion induced lower spike-specific IgGs with a lower neutralization titer than the same antigen cleaved of its Fc-portion (41), despite the fact that human IgG4 antibodies have the potential to interact with murine FcγRI (42). More importantly, Fc-fusion proteins have been extensively investigated as therapeutics due to the prolonged half-life via FcRn recycling, which led to the approval of 13 Fc-fusion biopharmaceutical drugs by the Europe Medicines Agency and the American Food and Drug Administration (3, 4, 43). Furthermore, recent advances in the Hemophilia A research field described that conjugating the coagulation factor VIII (FVIII) to a human IgG1 Fc-portion not only drastically improved the half-life of the drug, but also reduced the generation of anti-drug antibody towards FVIII by inducing a tolerogenic environment, hence providing a better protection against the hemorrhagic disorder (44, 45). Although mechanistic explanations are still under investigation, several reports have highlighted that the FVIII-Fc fusion protein leads to the induction of IL10-secreting T-regulatory cells and inhibits B-cell activation through the engagement of the inhibitory FcγRIIb thus reducing the secretion of FVIII-specific antibodies (46, 47).

The immunogenicity of RBD – Fc fusion proteins has also been analyzed recently (48). Immunization of BALB/c mice with DNA vaccines encoding either a head-to-tail RBD dimer or a dimerizing RBD – Fc fusion protein induced similar antibody responses (48). Since mutants of the Fc fragment impairing FcγR binding were not included it is difficult to dissect the effect of dimerization, half-life and differences in tertiary and quaternary structure on the antibody responses observed. Since the RBD antibody responses after immunization with RBD – Fc fusion proteins were similar for the LALA-PG mutant and the wild type Fc fragment our results suggest that dimerization or the prolonged half-life are the main determinants of the stronger antibody responses observed for some Fc-fusion proteins. Furthermore, we observed that neither FcγR binding nor the capability to recruit and activate the complement system resulted in higher RBD specific CD8+ T-cell responses or broader serum antibody neutralizing activities against SARS-CoV-2 variants.

In summary, our study showed in side-by-side experiments, that the effect of FcγR binding on the immunogenicity of Fc-fusion proteins depends on the protein the Fc fragment is fused to. Given the broad applications of Fc-fusion proteins as therapeutics and their potential as a vaccine platform, a better understanding of the determinants of these differential responses are needed to reduce the risk of anti-drug antibodies for therapeutic Fc fusion proteins and to unravel the benefits of Fc-fusion proteins in vaccine development.

## Limitation of the study

Although *i.m.* DNA electroporation offers the advantages to be highly immunogenic, to allow the expression of antigens endogenously, and to avoid contaminants from producer cells that are not removed during the purification processes, *in vivo* expression levels of the desired recombinant fusion protein are difficult to control. Therefore, the reduced *in vitro* expression level of the GASDIE mutants of our Fc-fusion proteins limit the conclusions that can be drawn from the use of this mutant. In addition, we cannot formally exclude, that different degrees of biotinylation of the different Fc-proteins affect the binding to FcγRs. However, since the biotinylation of the different Fc-fusion proteins were performed side-by-side for each of the two antigens by the same protocol and since the Fc-variants of each antigen-fusion protein only differ by a three amino acids, we consider it highly unlikely that the differences in FcγR binding observed (**Figure 4**) are due to differences in the degree of biotinylation. Consistent results for the RBD – Fc and gp120 – Fc mutants further argue against such a possibility. It should also be noted, that we did not determine the humoral immune response to the native form of the antigens in side-by-side experiments precluding any direct conclusions on the effect of fusing the Fc-portion to the antigens on their immunogenicity.

## Data availability statement

The original contributions presented in the study are included in the article/**Supplementary Files**, further inquiries can be directed to the corresponding authors.

## Ethics statement

The animal study was approved by the governmental authorities of Lower-Franconia under the license number 55.2-2532-2-1444. The study was conducted in accordance with the local legislation and institutional requirements.

## Author contributions

ER: Formal Analysis, Investigation, Writing – original draft, Visualization, Writing – review & editing. JW: Investigation, Writing – review & editing. SK: Investigation, Writing – review & editing. RD: Investigation, Writing – review & editing. VT: Conceptualization, Methodology, Project administration, Supervision, Writing – review & editing. KÜ: Conceptualization, Funding acquisition, Methodology, Supervision, Writing – review & editing, Project administration, Resources.

## References

[1] Czajkowsky DM Hu J Shao Z Pleass RJ. Fc-fusion proteins: new developments and future perspectives. EMBO Mol Med (2012) 4(10):1015–28. doi: 10.1002/emmm.201201379 PMC3491832 22837174

[2] Jafari R Zolbanin NM Rafatpanah H Majidi J Kazemi T. Fc-fusion proteins in therapy: an updated view. Curr Med Chem (2017) 24(12):1228–1237. doi: 10.2174/0929867324666170113112759 28088904

[3] Suzuki T Ishii-Watabe A Tada M Kobayashi T Kanayasu-Toyoda T Kawanishi T. Importance of neonatal fcR in regulating the serum half-life of therapeutic proteins containing the fc domain of human igG1: A comparative study of the affinity of monoclonal antibodies and fc-fusion proteins to human neonatal fcR. J Immunol (2010) 184(4):1968–76. doi: 10.4049/jimmunol.0903296 20083659

[4] Rath T Baker K Dumont JA Peters RT Jiang H Qiao SW. Fc-fusion proteins and FcRn: structural insights for longer-lasting and more effective therapeutics. Crit Rev Biotechnol (2015) 35(2):235–54. doi: 10.3109/07388551.2013.834293 PMC4876602 24156398

[5] Junker F Gordon J Qureshi O. Fc gamma receptors and their role in antigen uptake, presentation, and T cell activation. Front Immunol (2020) 11. doi: 10.3389/fimmu.2020.01393 PMC7350606 32719679

[6] Levin D Golding B Strome SE Sauna ZE. Fc fusion as a platform technology: potential for modulating immunogenicity. Trends Biotechnol (2015) 33(1):27–34. doi: 10.1016/j.tibtech.2014.11.001 25488117

[7] PLOTKIN SA. Immunologic correlates of protection induced by vaccination. Pediatr Infect Dis J (2001) 20(1):63–75. doi: 10.1097/00006454-200101000-00013 11176570

[8] Plotkin SA. Correlates of protection induced by vaccination. Clin Vaccine Immunol (2010) 17(7):1055–65. doi: 10.1128/CVI.00131-10 PMC2897268 20463105

[9] Shubin Z Li W Poonia B Ferrari G LaBranche C Montefiori D. An HIV envelope gp120-fc fusion protein elicits effector antibody responses in rhesus macaques. Clin Vaccine Immunol (2017) 24(6). doi: 10.1128/CVI.00028-17 PMC5461376 28404572

[10] Zhao B Zhang X Krummenacher C Song S Gao L Zhang H. Immunization with fc-based recombinant epstein–barr virus gp350 elicits potent neutralizing humoral immune response in a BALB/c mice model. Front Immunol (2018) 9. doi: 10.3389/fimmu.2018.00932 PMC5938345 29765376

[11] Lamers MM Haagmans BL. SARS-coV-2 pathogenesis. Nat Rev Microbiol (2022) 20(5):270–84. doi: 10.1038/s41579-022-00713-0 35354968

[12] Jackson CB Farzan M Chen B Choe H. Mechanisms of SARS-CoV-2 entry into cells. Nat Rev Mol Cell Biol (2022) 23(1):3–20. doi: 10.1038/s41580-021-00418-x 34611326 PMC8491763

[13] Hurt AC Wheatley AK. Neutralizing antibody therapeutics for COVID-19. Viruses (2021) 13(4):628. doi: 10.3390/v13040628 33916927 PMC8067572

[14] Huang Y Sun H Yu H Li S Zheng Q Xia N. Neutralizing antibodies against SARS-CoV-2: current understanding, challenge and perspective. Antib Ther (2020) 3(4):285–99. doi: 10.1093/abt/tbaa028 PMC7799234 33912797

[15] Liu Y Arase H. Neutralizing and enhancing antibodies against SARS-CoV-2. Inflammation Regen. (2022) 42(1):58. doi: 10.1186/s41232-022-00233-7 PMC9720987 36471381

[16] Sun S He L Zhao Z Gu H Fang X Wang T. Recombinant vaccine containing an RBD-Fc fusion induced protection against SARS-CoV-2 in nonhuman primates and mice. Cell Mol Immunol (2021) 18(4):1070–3. doi: 10.1038/s41423-021-00658-z PMC7966917 33731916

[17] Liu Z Xu W Xia S Gu C Wang X Wang Q. RBD-Fc-based COVID-19 vaccine candidate induces highly potent SARS-CoV-2 neutralizing antibody response. Signal Transduct Target Ther (2020) 5(1):282. doi: 10.1038/s41392-020-00402-5 33247109 PMC7691975

[18] Hansen J Baum A Pascal KE Russo V Giordano S Wloga E. Studies in humanized mice and convalescent humans yield a SARS-CoV-2 antibody cocktail. Sci (1979). (2020) 369(6506):1010–4. doi: 10.1126/science.abd0827 PMC7299284 32540901

[19] Zhou C Zai X Zhou Z Li R Zhang Y Li Y. RBD206-sc-dimer induced robust cross-neutralization against SARS-CoV-2 and variants of concern. Signal Transduct Target Ther (2021) 6(1):390. doi: 10.1038/s41392-021-00798-8 34759271 PMC8578908

[20] Dai L Zheng T Xu K Han Y Xu L Huang E. A universal design of betacoronavirus vaccines against COVID-19, MERS, and SARS. Cell (2020) 182(3):722–733.e11. doi: 10.1016/j.cell.2020.06.035 32645327 PMC7321023

[21] An Y Li S Jin X Han Jb Xu K Xu S. A tandem-repeat dimeric RBD protein-based covid-19 vaccine zf2001 protects mice and nonhuman primates. Emerg Microbes Infect (2022) 11(1):1058–71. doi: 10.1080/22221751.2022.2056524 PMC9009945 35311493

[22] Kothe DL Decker JM Li Y Weng Z Bibollet-Ruche F Zammit KP. Antigenicity and immunogenicity of HIV-1 consensus subtype B envelope glycoproteins. Virology (2007) 360(1):218–34. doi: 10.1016/j.virol.2006.10.017 PMC1945152 17097711

[23] Nguyen HNP Steede NK Robinson JE Landry SJ. Conformational instability governed by disulfide bonds partitions the dominant from subdominant helper T-cell responses specific for HIV-1 envelope glycoprotein gp120. Vaccine (2015) 33(25):2887–96. doi: 10.1016/j.vaccine.2015.04.082 PMC4443875 25944298

[24] Dispinseri S Secchi M Pirillo MF Tolazzi M Borghi M Brigatti C. Neutralizing antibody responses to SARS-CoV-2 in symptomatic COVID-19 is persistent and critical for survival. Nat Commun (2021) 12(1):2670. doi: 10.1038/s41467-021-22958-8 33976165 PMC8113594

[25] Zech F Schniertshauer D Jung C Herrmann A Cordsmeier A Xie Q. Spike residue 403 affects binding of coronavirus spikes to human ACE2. Nat Commun (2021) 12(1):6855. doi: 10.1038/s41467-021-27180-0 34824253 PMC8617078

[26] Stewart R Hammond SA Oberst M Wilkinson RW. The role of Fc gamma receptors in the activity of immunomodulatory antibodies for cancer. J Immunother Cancer. (2014) 2(1):29. doi: 10.1186/s40425-014-0029-x

[27] Bruhns P Jönsson F. Mouse and human FcR effector functions. Immunol Rev (2015) 268(1):25–51. doi: 10.1111/imr.12350 26497511

[28] Lo M Kim HS Tong RK Bainbridge TW Vernes JM Zhang Y. Effector-attenuating substitutions that maintain antibody stability and reduce toxicity in mice. J Biol Chem (2017) 292(9):3900–8. doi: 10.1074/jbc.M116.767749 PMC5339770 28077575

[29] Schlothauer T Herter S Koller CF Grau-Richards S Steinhart V Spick C. Novel human IgG1 and IgG4 Fc-engineered antibodies with completely abolished immune effector functions. Protein Eng Design Selection. (2016) 29(10):457–66. doi: 10.1093/protein/gzw040 27578889

[30] Moldt B Schultz N Dunlop DC Alpert MD Harvey JD Evans DT. A Panel of IgG1 b12 Variants with Selectively Diminished or Enhanced Affinity for Fcγ Receptors To Define the Role of Effector Functions in Protection against HIV. J Virol (2011) 85(20):10572–81. doi: 10.1128/JVI.05541-11 PMC3187489 21849450

[31] Heß R Lapuente D Maaske A Kirschning C Ruland J Lepenies B. Glycosylation of HIV env impacts igG subtype responses to vaccination. Viruses (2019) 11(2):153. doi: 10.3390/v11020153 30781796 PMC6410111

[32] Dormeshkin D Katsin M Stegantseva M Golenchenko S Shapira M Dubovik S. Design and immunogenicity of SARS-coV-2 DNA vaccine encoding RBD-PVXCP fusion protein. Vaccines (Basel). (2023) 11(6):1014. doi: 10.3390/vaccines11061014 37376403 PMC10300735

[33] Jeong H Choi YM Seo H Kim BJ. A novel DNA vaccine against SARS-coV-2 encoding a chimeric protein of its receptor-binding domain (RBD) fused to the amino-terminal region of hepatitis B virus preS1 with a W4P mutation. Front Immunol (2021) 12. doi: 10.3389/fimmu.2021.637654 PMC7959807 33732258

[34] Brady JM Phelps M MacDonald SW Lam EC Nitido A Parsons D. Antibody-mediated prevention of vaginal HIV transmission is dictated by IgG subclass in humanized mice. Sci Transl Med (2022) 14(655). doi: 10.1126/scitranslmed.abn9662 PMC9507259 35895834

[35] Bournazos S Klein F Pietzsch J Seaman MS Nussenzweig MC Ravetch JV. Broadly neutralizing anti-HIV-1 antibodies require fc effector functions for *in vivo* activity. Cell (2014) 158(6):1243–53. doi: 10.1016/j.cell.2014.08.023 PMC4167398 25215485

[36] Hessell AJ Hangartner L Hunter M Havenith CEG Beurskens FJ Bakker JM. Fc receptor but not complement binding is important in antibody protection against HIV. Nature (2007) 449(7158):101–4. doi: 10.1038/nature06106 17805298

[37] Fantuzzi L Purificato C Donato K Belardelli F Gessani S. Human immunodeficiency virus type 1 gp120 induces abnormal maturation and functional alterations of dendritic cells: a novel mechanism for AIDS pathogenesis. J Virol (2004) 78(18):9763–72. doi: 10.1128/JVI.78.18.9763-9772.2004 PMC515003 15331709

[38] Shan M Klasse PJ Banerjee K Dey AK Iyer SPN Dionisio R. HIV-1 gp120 mannoses induce immunosuppressive responses from dendritic cells. PloS Pathog (2007) 3(11):e169. doi: 10.1371/journal.ppat.0030169 17983270 PMC2048530

[39] Chougnet C Gessani S. Role of gp120 in dendritic cell dysfunction in HIV infection. J Leukoc Biol (2006) 80(5):994–1000. doi: 10.1189/jlb.0306135 16912071

[40] Chen H Xu X Jones IM. Immunogenicity of the outer domain of a HIV-1 clade C gp120. Retrovirology (2007) 4(1):33. doi: 10.1186/1742-4690-4-33 17509143 PMC1891314

[41] Chun J Cho Y Park KH Choi H Cho H Lee HJ. Effect of fc fusion on folding and immunogenicity of middle east respiratory syndrome coronavirus spike protein. J Microbiol Biotechnol (2019) 29(5):813–9. doi: 10.4014/jmb.1903.03043 30982320

[42] Dekkers G Bentlage AEH Stegmann TC Howie HL Lissenberg-Thunnissen S Zimring J. Affinity of human IgG subclasses to mouse Fc gamma receptors. MAbs (2017) 9(5):767–73. doi: 10.1080/19420862.2017.1323159 PMC5524164 28463043

[43] Duivelshof BL Murisier A Camperi J Fekete S Beck A Guillarme D. Therapeutic Fc-fusion proteins: Current analytical strategies. J Sep Sci (2021) 44(1):35–62. doi: 10.1002/jssc.202000765 32914936

[44] Cormier M Batty P Tarrant J Lillicrap D. Advances in knowledge of inhibitor formation in severe haemophilia A. Br J Haematol (2020) 189(1):39–53. doi: 10.1111/bjh.16377 32064603

[45] Blumberg RS Lillicrap D. Tolerogenic properties of the Fc portion of IgG and its relevance to the treatment and management of hemophilia. Blood (2018) 131(20):2205–14. doi: 10.1182/blood-2017-12-822908 PMC5958656 29588277

[46] Georgescu MT Moorehead PC Liu T Dumont J Scott DW Hough C. Recombinant factor VIII fc inhibits B cell activation via engagement of the fcγRIIB receptor. Front Immunol (2020) 11. doi: 10.3389/fimmu.2020.00138 PMC7025534 32117285

[47] Krishnamoorthy S Liu T Drager D Patarroyo-White S Chhabra ES Peters R. Recombinant factor VIII Fc (rFVIIIFc) fusion protein reduces immunogenicity and induces tolerance in hemophilia A mice. Cell Immunol (2016) 301:30–9. doi: 10.1016/j.cellimm.2015.12.008 PMC4936482 26775174

[48] Zhang T Wang Z Yang J Xu X. Immunogenicity of novel DNA vaccines encoding receptor-binding domain (RBD) dimer-Fc fusing antigens derived from different SARS-CoV-2 variants of concern. J Med Virol (2023) 95(2). doi: 10.1002/jmv.28563 36755368

