# Supplementary material for: Antigen-dependent modulation of immune responses to antigen-Fc fusion proteins by Fc-effector functions
Source: Front Immunol. 2023 Oct 5;14:1275193. doi: 10.3389/fimmu.2023.1275193 (PMC10585040; doi:10.3389/fimmu.2023.1275193)
Supplement: Supplementary file 1 [file Presentation_1.pptx]

## Slide 1
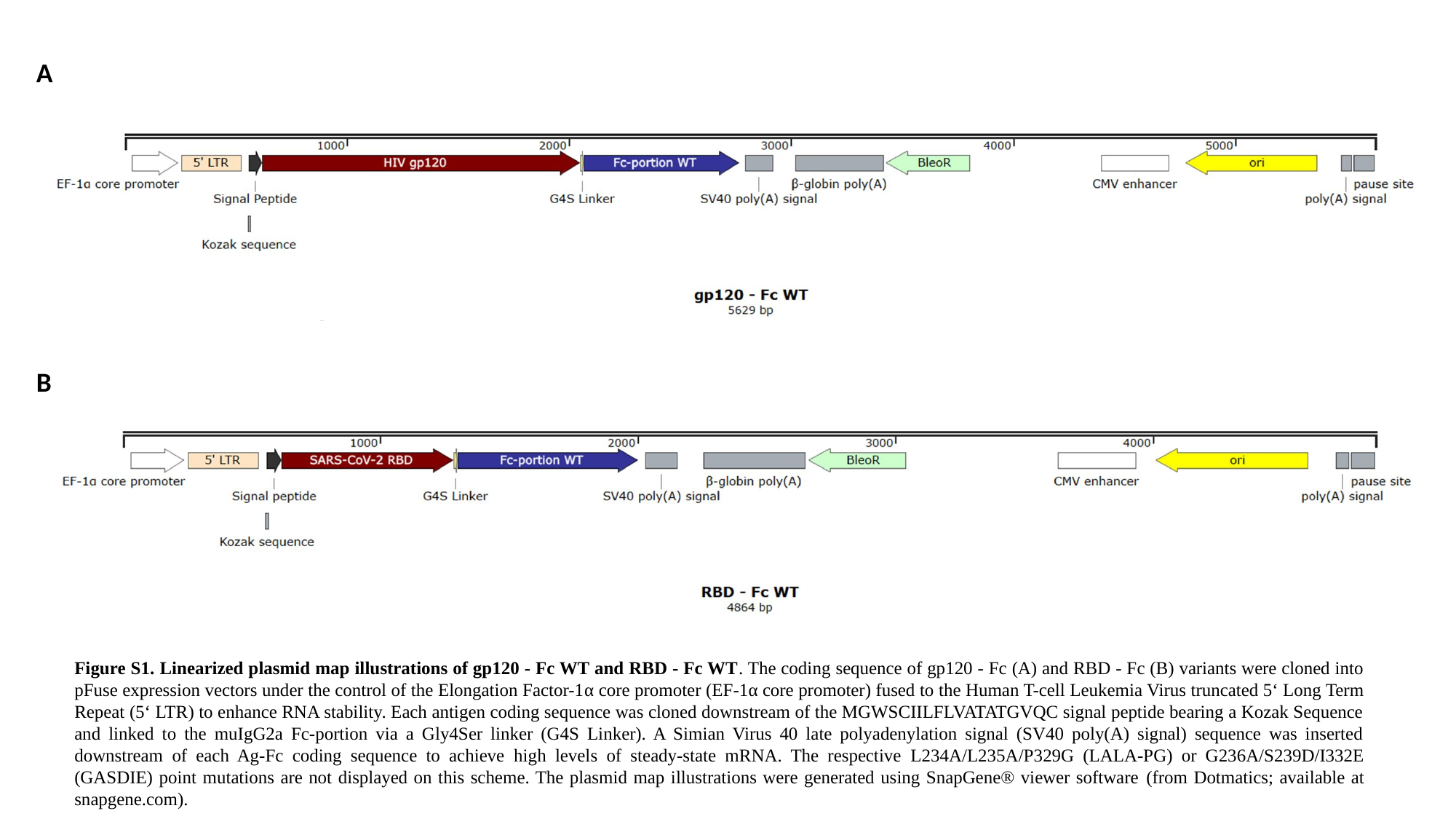

A
B
Figure S1. Linearized plasmid map illustrations of gp120 - Fc WT and RBD - Fc WT. The coding sequence of gp120 - Fc (A) and RBD - Fc (B) variants were cloned into pFuse expression vectors under the control of the Elongation Factor-1α core promoter (EF-1α core promoter) fused to the Human T-cell Leukemia Virus truncated 5‘ Long Term Repeat (5‘ LTR) to enhance RNA stability. Each antigen coding sequence was cloned downstream of the MGWSCIILFLVATATGVQC signal peptide bearing a Kozak Sequence and linked to the muIgG2a Fc-portion via a Gly4Ser linker (G4S Linker). A Simian Virus 40 late polyadenylation signal (SV40 poly(A) signal) sequence was inserted downstream of each Ag-Fc coding sequence to achieve high levels of steady-state mRNA. The respective L234A/L235A/P329G (LALA-PG) or G236A/S239D/I332E (GASDIE) point mutations are not displayed on this scheme. The plasmid map illustrations were generated using SnapGene® viewer software (from Dotmatics; available at snapgene.com).

## Slide 2
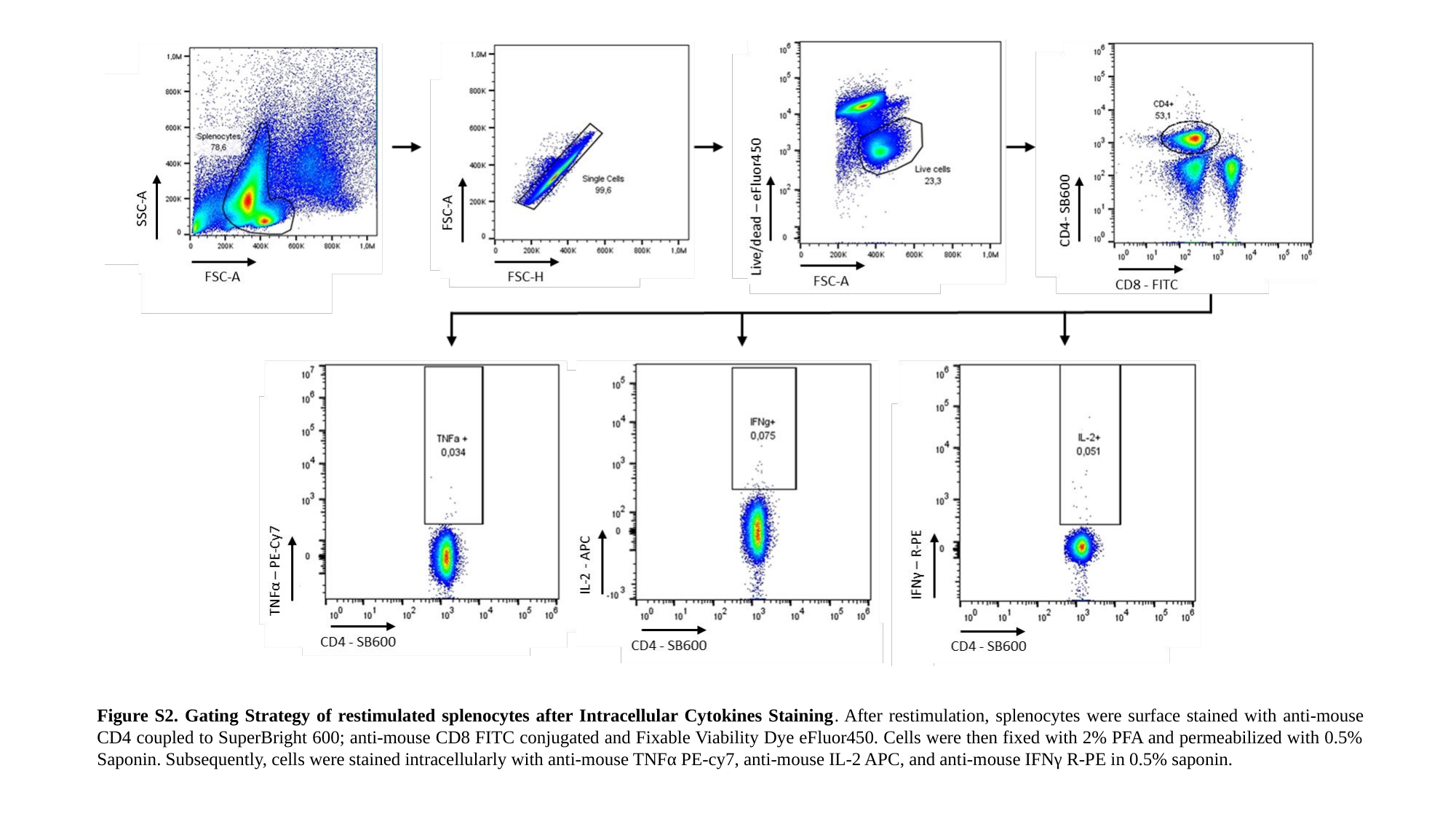

Figure S2. Gating Strategy of restimulated splenocytes after Intracellular Cytokines Staining. After restimulation, splenocytes were surface stained with anti-mouse CD4 coupled to SuperBright 600; anti-mouse CD8 FITC conjugated and Fixable Viability Dye eFluor450. Cells were then fixed with 2% PFA and permeabilized with 0.5% Saponin. Subsequently, cells were stained intracellularly with anti-mouse TNFα PE-cy7, anti-mouse IL-2 APC, and anti-mouse IFNγ R-PE in 0.5% saponin.

## Slide 3
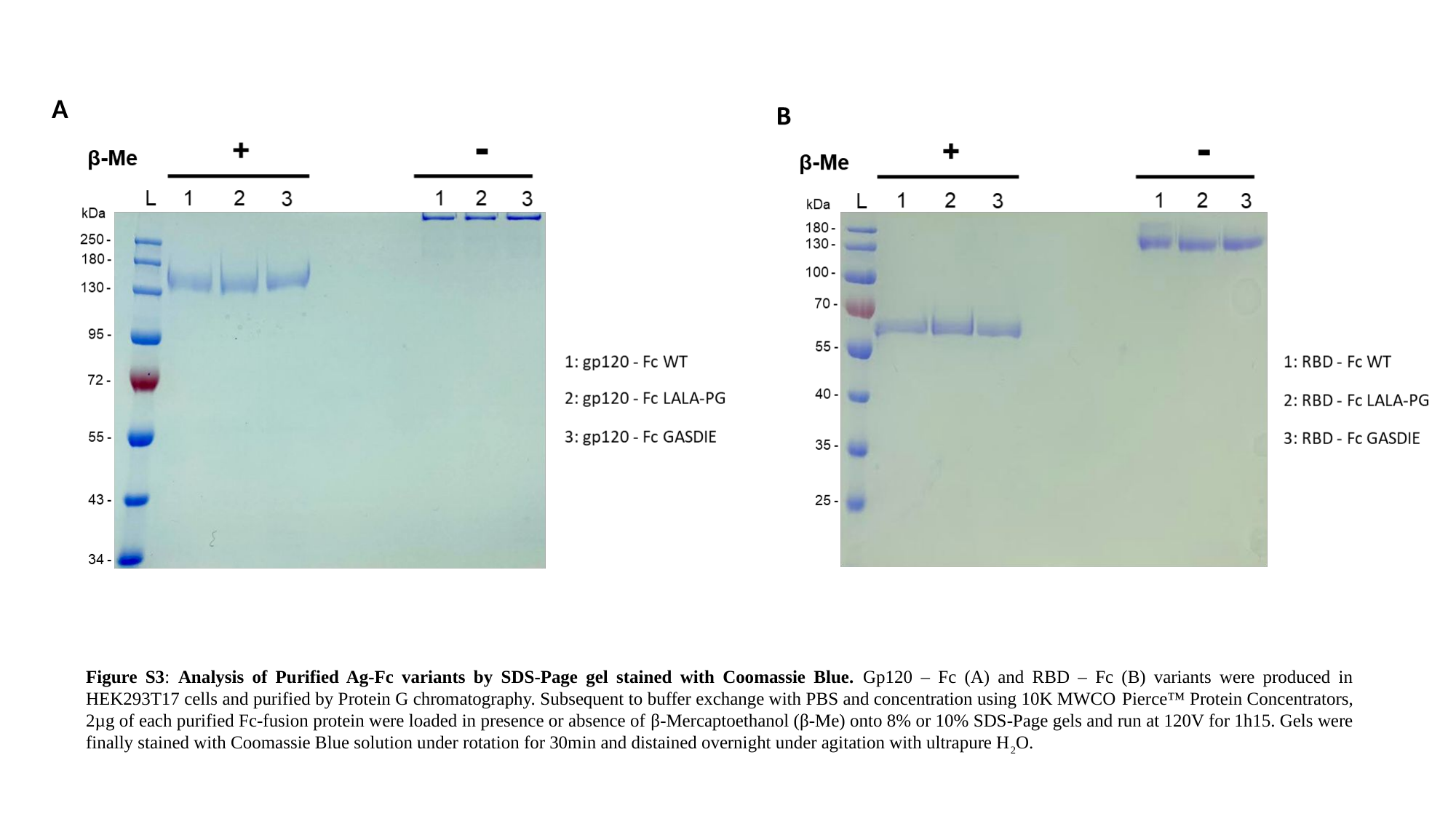

A
Figure S3: Analysis of Purified Ag-Fc variants by SDS-Page gel stained with Coomassie Blue. Gp120 – Fc (A) and RBD – Fc (B) variants were produced in HEK293T17 cells and purified by Protein G chromatography. Subsequent to buffer exchange with PBS and concentration using 10K MWCO Pierce™ Protein Concentrators, 2µg of each purified Fc-fusion protein were loaded in presence or absence of β-Mercaptoethanol (β-Me) onto 8% or 10% SDS-Page gels and run at 120V for 1h15. Gels were finally stained with Coomassie Blue solution under rotation for 30min and distained overnight under agitation with ultrapure H2O.

## Slide 4
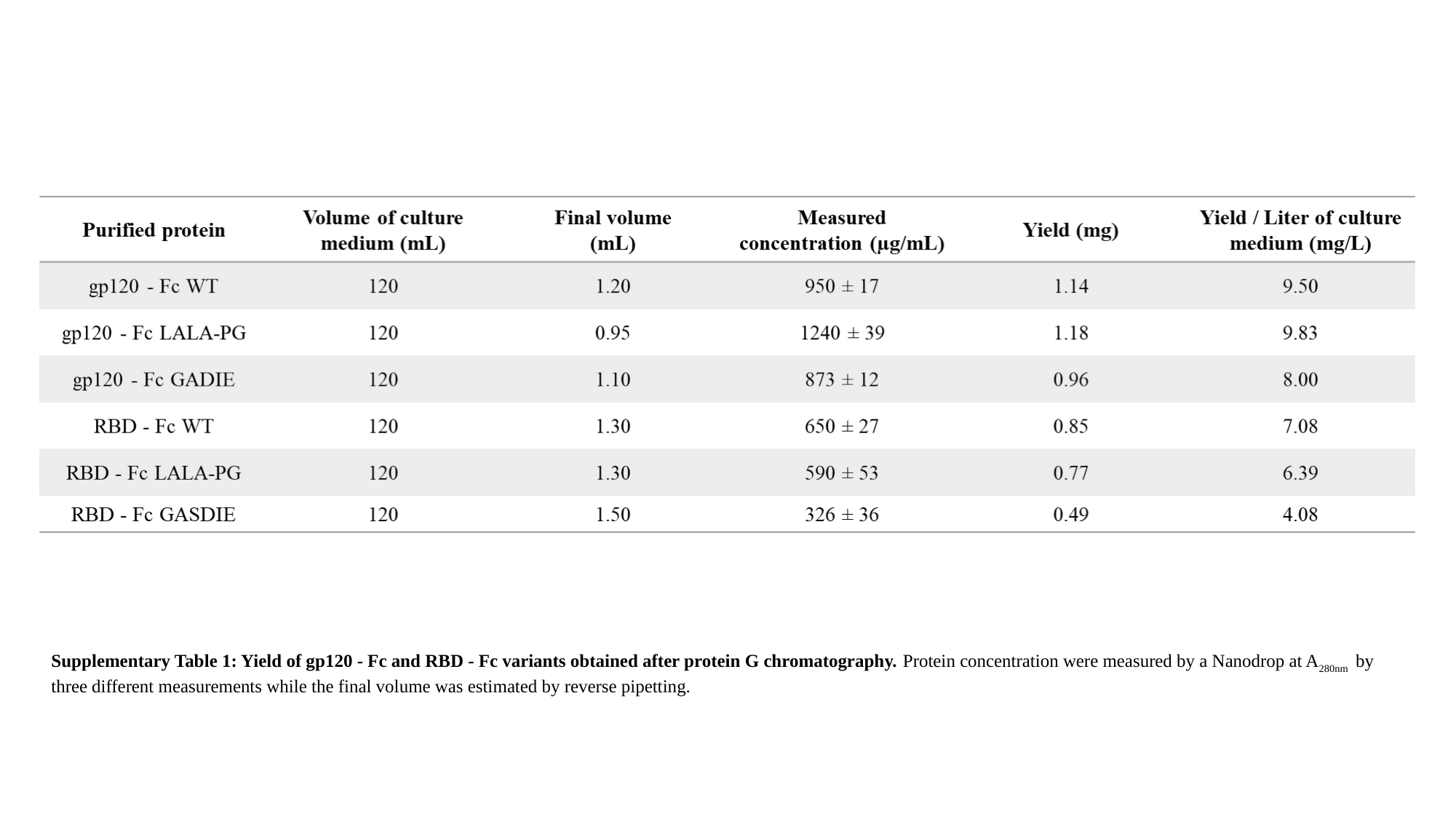

Supplementary Table 1: Yield of gp120 - Fc and RBD - Fc variants obtained after protein G chromatography. Protein concentration were measured by a Nanodrop at A280nm by three different measurements while the final volume was estimated by reverse pipetting.

## Slide 5
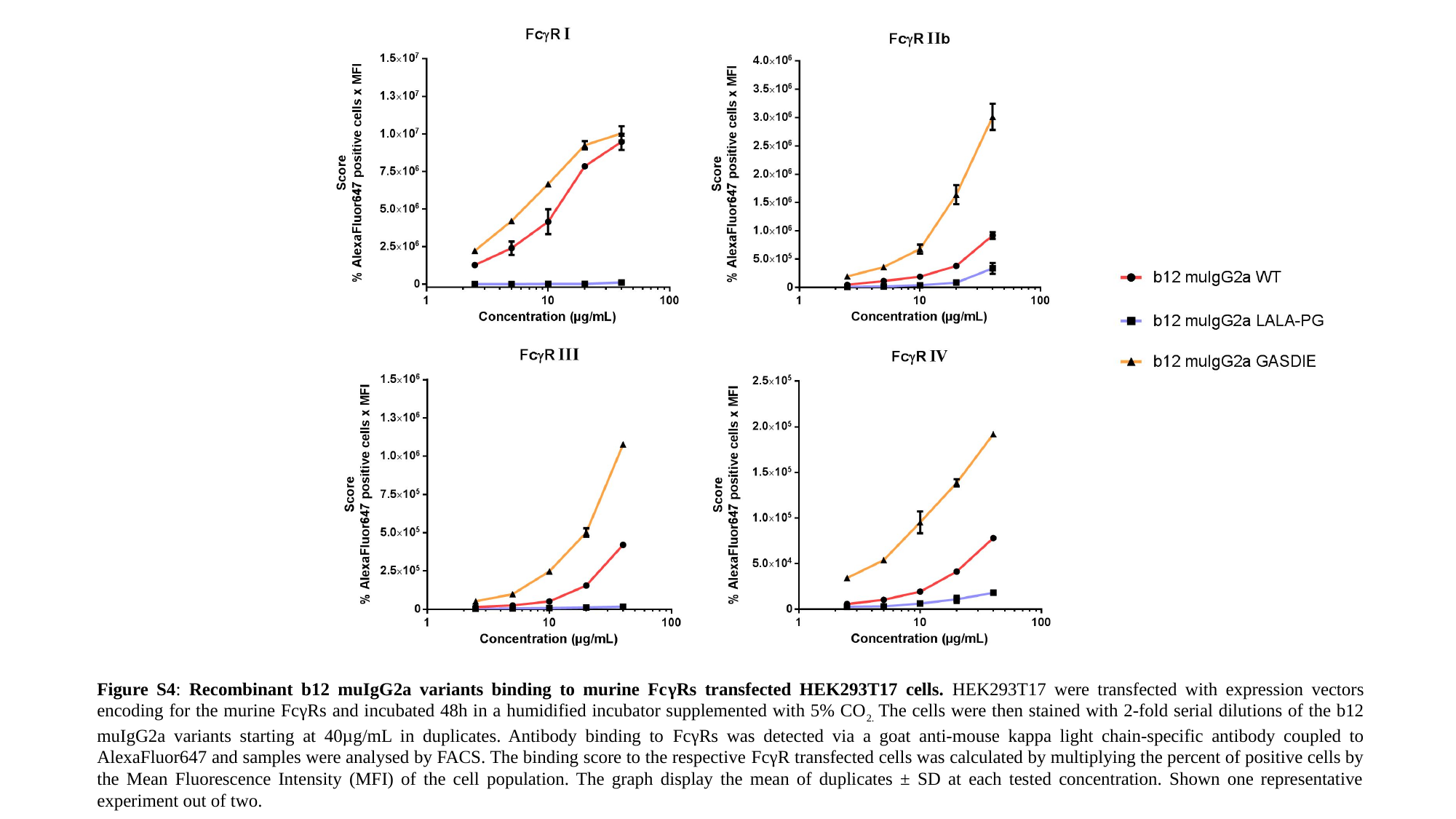

Figure S4: Recombinant b12 muIgG2a variants binding to murine FcγRs transfected HEK293T17 cells. HEK293T17 were transfected with expression vectors encoding for the murine FcγRs and incubated 48h in a humidified incubator supplemented with 5% CO2. The cells were then stained with 2-fold serial dilutions of the b12 muIgG2a variants starting at 40µg/mL in duplicates. Antibody binding to FcγRs was detected via a goat anti-mouse kappa light chain-specific antibody coupled to AlexaFluor647 and samples were analysed by FACS. The binding score to the respective FcγR transfected cells was calculated by multiplying the percent of positive cells by the Mean Fluorescence Intensity (MFI) of the cell population. The graph display the mean of duplicates ± SD at each tested concentration. Shown one representative experiment out of two.

## Slide 6
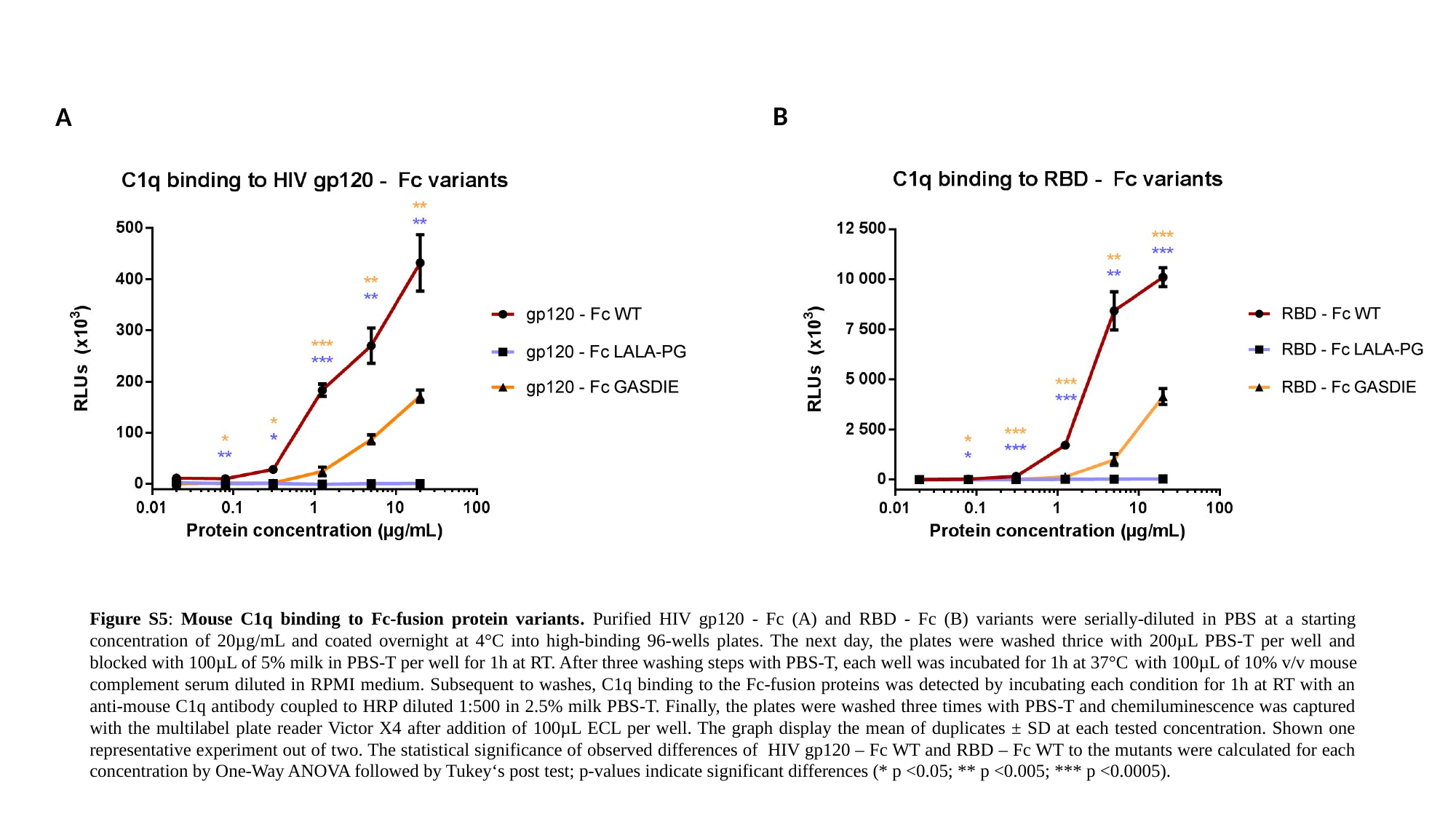

B
A
Figure S5: Mouse C1q binding to Fc-fusion protein variants. Purified HIV gp120 - Fc (A) and RBD - Fc (B) variants were serially-diluted in PBS at a starting concentration of 20µg/mL and coated overnight at 4°C into high-binding 96-wells plates. The next day, the plates were washed thrice with 200µL PBS-T per well and blocked with 100µL of 5% milk in PBS-T per well for 1h at RT. After three washing steps with PBS-T, each well was incubated for 1h at 37°C with 100µL of 10% v/v mouse complement serum diluted in RPMI medium. Subsequent to washes, C1q binding to the Fc-fusion proteins was detected by incubating each condition for 1h at RT with an anti-mouse C1q antibody coupled to HRP diluted 1:500 in 2.5% milk PBS-T. Finally, the plates were washed three times with PBS-T and chemiluminescence was captured with the multilabel plate reader Victor X4 after addition of 100µL ECL per well. The graph display the mean of duplicates ± SD at each tested concentration. Shown one representative experiment out of two. The statistical significance of observed differences of HIV gp120 – Fc WT and RBD – Fc WT to the mutants were calculated for each concentration by One-Way ANOVA followed by Tukey‘s post test; p-values indicate significant differences (* p <0.05; ** p <0.005; *** p <0.0005).
